# Evaluation of a Cell-Adapted Live Attenuated African Swine Fever Virus Thai-Strain Vaccine Candidate: Highlighting Enhanced Virulence Risk in Co-Infected Pigs

**DOI:** 10.3390/vaccines13121189

**Published:** 2025-11-24

**Authors:** Challika Kaewborisuth, Theeradej Thaweerattanasinp, Nanchaya Wanasen, Apidsada Chorpunkul, Payuda Hansoongnern, Nathiphat Tanwattana, Kanjana Srisutthisamphan, Janya Saenboonrueng, Asawin Wanitchang, Suphot Wattanaphansak, Rachod Tantilertcharoen, Nattachai Suksawat, Jarin Kramyu, Benjamas Liwnaree, Papon Muangsanit, Kriangkrai Chaikhum, Tapanut Songkasupa, Thitawat Chanthaworn, Anan Jongkaewwattana

**Affiliations:** 1 Virology and Vaccine Technology Research Team, National Center for Genetic Engineering and Biotechnology (BIOTEC), National Science and Technology Development Agency (NSTDA), Pathum Thani 12120, Thailand; theeradej.tha@biotec.or.th (T.T.); nanchaya.wan@biotec.or.th (N.W.); apidsada.cho@ncr.nstda.or.th (A.C.); payuda.han@biotec.or.th (P.H.); nathiphat.tan@ncr.nstda.or.th (N.T.); kanjana.sri@biotec.or.th (K.S.); janya@biotec.or.th (J.S.); asawin.wan@biotec.or.th (A.W.); nattachai.suk@ncr.nstda.or.th (N.S.); jarin@biotec.or.th (J.K.); benjamas.chu@biotec.or.th (B.L.); papon.mua@biotec.or.th (P.M.); anan.jon@biotec.or.th (A.J.); 2 Veterinary Diagnostic Laboratory, Department of Veterinary Medicine, Faculty of Veterinary Science, Chulalongkorn University, Bangkok 10330, Thailand; supot.w@chula.ac.th (S.W.); rachod.t@chula.ac.th (R.T.); 3 Veterinary Biologics Assay and Research Center, National Institute of Animal Health, Department of Livestock Development, Nakhonratchasima 30130, Thailand; nuengvet1@gmail.com (K.C.); thitawatana@hotmail.com (T.C.); 4 National Institute of Animal Health, Department of Livestock Development, Bangkok 10900, Thailand; tapanut.s@dld.go.th

**Keywords:** African swine fever virus, live attenuated vaccine, cell adaptation, vaccine efficacy, vaccine safety, latent infections

## Abstract

Background/Objectives: African swine fever (ASF) is a devastating disease affecting the swine industry globally. Development of safe and effective vaccines is an urgent need. This study aimed to evaluate, caASFV001-MA52, a cell-adapted ASFV strain derived from serial passaging in MA-104 cells, as a promising live-attenuated vaccine (LAV) candidate against virulent ASFV infection. Methods: Seven-week-old, crossbred pigs were immunized with caASFV001-MA52 (at a dose of 10^5^ TCID_50_) and subsequently challenged with a lethal dose of virulent ASFV. Vaccine efficacy was measured through clinical monitoring, immunological and virological analyses, and pathological assessments of tissue protection and viral load reduction. Safety was critically assessed, particularly regarding its profile in animals with concurrent endemic porcine infections, including PCV, PRRSV and *S. suis*. Results: caASFV001-MA52 exhibits partial protection (70–80%) against the lethal challenge. Vaccinated and challenged survivors exhibited reduced viral loads and significantly alleviated pathological lesions compared to controls. Safety evaluations revealed that the vaccine’s profile is susceptible to concurrent infection. Pigs co-infected with endemic porcine pathogens showed increased virulence and mortality following vaccination. Although vaccination temporarily reactivated latent viral infections (PCV2, PCV3, and PRRSV), most surviving pigs effectively controlled and eliminated these co-infections. Conclusions: The caASFV001-MA52 strain demonstrates promising immunogenicity and protection against lethal challenges, supporting its continued development as an LAV candidate. However, the observed safety concerns regarding concurrent infections emphasize the critical need for veterinary health surveillance during its future practical application.

## 1. Introduction

African swine fever (ASF), a highly contagious and devastating disease that affects both domestic and wild pigs, is caused by the African swine fever virus (ASFV), a large, complex double-stranded DNA virus in the *Asfarviridae* family [1]. It causes an acute hemorrhagic fever with mortality approaching 100%, threatening global swine production and food security. Spread of the highly virulent genotype II strain across Africa, Europe, Asia, and the Americas has resulted in millions of animal losses, affecting pork supply chains, market prices, and international trade [2,3,4]. In Thailand, the ASF outbreak had a significant impact on the swine industry, leading to a 43.35% reduction in swine raisers, particularly affecting small-scale farms. The outbreak also caused a sharp increase in pork prices, making meat less affordable for low-income households [3]. The economic consequences were severe, with disruptions in pork production and trade affecting both local and national markets. The control of ASF presents considerable challenges due to the limited availability of safe and widely effective vaccines and the lack of targeted antiviral treatments. Consequently, containment strategies rely on strict biosecurity measures, rapid diagnostics, mass culling of infected and exposed animals, and movement restrictions. These approaches impose substantial economic and logistical burdens on the swine industry [5,6]. Their effectiveness is insufficient to achieve widespread eradication in complex epidemiological settings, thereby emphasizing the critical need for an effective and safe vaccine to provide a sustainable and long-term solution.

Attempts to develop ASFV vaccines have been hindered by ASFV’s complex structure, immune evasion mechanisms, and an incomplete understanding of the correlates of protective immunity [7,8,9], complicating vaccine design. Among the vaccine approaches, live-attenuated vaccines (LAVs) derived from naturally attenuated strains or targeted genetic modifications have shown the most promising strategy, which induces robust and long-lasting protection [10]. While certain experimental LAVs such as ASFV-G-ΔI177L and ASFV-G-∆MGF vaccine strains have shown protective efficacy [11,12,13,14], cases of reversion to virulence, risk of horizontal transmission, and an emergence of vaccine-like variants after vaccination raise concerns about long-term safety [15,16]. Safety concerns, including risks of residual virulence, potential reversion to virulence, and the establishment of carrier states in vaccinated animals, complicate their widespread commercial application and necessitate a careful balance between attenuation and immunogenicity [8,17]. Another promising strategy for producing safer and scalable LAVs involves cell adaptation through serial passage in specific cell lines [18,19]. This approach facilitates viral attenuation via accumulated genetic modifications, such as gene deletions or mutations that reduce virulence while preserving immunogenicity [20,21]. Additionally, propagation in established immortalized cell lines offers advantages for large-scale vaccine production compared to primary cell cultures such as porcine alveolar macrophages (PAMs), which pose challenges in standardization and scalability. The experimental cell-adapted strain ASFV-MEC-01, generated through serial passage in CA-CAS-01-A cells and characterized by multiple gene deletions, demonstrated complete protection against a lethal ASFV challenge in pigs, reinforcing the potential of this strategy [20]. A good balance between safety and protective efficacy is a central challenge in the development of cell-adapted live attenuated African swine fever virus (caASFV) vaccines since over-attenuation can lead to poor protection, while under-attenuation can cause disease. Critically, the overall safety of current LAV candidates is primarily assessed in specific-pathogen-free (SPF) pigs, but their performance and safety profile in commercial settings, where pigs frequently harbor latent viral and bacterial co-infections, remain largely unknown.

Given the potential of a cell-adapted ASFV candidate, we described a cell-adapted ASFV strain derived from MA-104 cells (caASFV001-MA) that exhibited significant genetic changes, including deletions in multigene family (MGF) regions within the left variable region (LVR) and right variable region (RVR), which are associated with viral immune evasion and host–pathogen interactions [22]. Here, we aimed to assess the efficacy and safety of the cell-adapted ASFV vaccine candidate, caASFV001-MA52. Specifically, we evaluated its ability to induce protective immunity against a virulent challenge and measured its impact on survival, pathological damage, and viral load, presenting in co-infected pigs.

## 2. Materials and Methods

### 2.1. Cells and Viruses

The caASFV001-MA strain [22] was serially passaged in sub-confluent monolayers of MA-104 cells (ATCC CRL-2378.1) in T75 flasks. After a 3 h incubation at 37 °C and 5% CO_2_, cells were washed with phosphate-buffered saline (PBS) and replenished with Dulbecco’s Modified Eagle Medium (DMEM) containing 10% fetal bovine serum (FBS) and incubated for 6 days before harvest. Upon reaching passage 52 (caASFV001-MA52), virus-containing cell suspensions underwent three freeze–thaw cycles, followed by centrifugation at 5000× *g* for 10 min to remove debris. Supernatants were harvested and filtered through 0.45-μm syringe filters. Virus stocks and virus doses were titrated in MA-104 cells by TCID_50_ assay using the Reed and Muench method [23]. ASFV DNA was extracted for whole genome sequencing as described previously [22].

The virulent ASFV strain (ASFV/GII/Thailand/NIAH-CR-19/2022) used for virus challenge was isolated from a pig with severe hemorrhage in Thailand in 2022. Virus isolation and titration were conducted using macrophages derived from porcine peripheral blood mononuclear cells (PBMCs), which were collected from a clinically healthy donor pigs confirmed to be free of porcine circovirus 2 (PCV2) and PCV3, porcine reproductive and respiratory syndrome virus (PRRSV; EU, US, HP strains), classical swine fever virus (CSFV), and ASFV. PBMCs were prepared from defibrinated pig blood as previously described [24]. Briefly, the cells were cultured in Roswell Park Memorial Institute (RPMI) 1640 medium (Thermo Fisher Scientific, Waltham, MA, USA) containing 30% autogenous pig serum and supplemented with an antibiotic–antimycotic solution (Thermo Fisher Scientific). For titrations, cells were seeded in 96-well culture plates at 100 μL/well at a density of 1.5 × 10^6^ cells/mL and cultivated for 72 h. Then, each well was inoculated with 100 μL of serially diluted samples (10^−1^ to 10^−8^) prepared in cell culture medium for endpoint titration. After 24 h, 20 μL of PBS containing 1% pig erythrocytes from the same donor pig were added. Infected macrophages were examined for erythrocyte attachment. Virus titers were calculated using the Reed and Muench method to determine the 50% hemadsorption dose. Virus stocks (at passage 5) were titrated in PBMC culture and stored in aliquots at −80 °C until use.

### 2.2. Animals and Disease Screening

Because commercial specific-pathogen-free (SPF) pigs are not available in Thailand, a strict pathogen screening process was performed for the present study. Blood samples were collected from 5-week-old pigs and analyzed by quantitative PCR (qPCR) for viral and bacterial pathogens, including PCV2, PCV3, PRRSV, CSFV, ASFV, *Mycoplasma hyorhinis* (*Mhr*), *M. hyopneumoniae* (*Mhp*), and *Streptococcus suis* (*S. suis*). All animals used were genetically uniform crossbred males (Landrace × Large White × Duroc) sourced from the same farm and the same rearing batch.

In the initial study, we confirmed that all pigs were negative for PRRSV. The cohort showed varied positivity for other pathogens, including PCV2, PCV3, *Mhr,* and *Mhp* (Table S1). Only those animals that tested negative for all screened pathogens were separated and retested to confirm their specific pathogen-free-like status for the study. All selected pigs were confirmed negative for ASFV-specific antibodies using the ID Screen^®^ African Swine Fever Indirect ELISA Kit (Innovative Diagnostics, Grabels, France) (Table S2), thereby validating their suitability for inclusion in the experiment. The pigs were then transported to the Veterinary Biologics Assay and Research Center (VBAC), Department of Livestock Development (DLD). The experiments started following a seven-day quarantine period to ensure the stability of the animals’ health status. Throughout the experiment (both post-vaccination and post-challenge phases), individual pigs were maintained in high-security containment units to prevent cross-contamination and environmental release. Pigs were fed commercial swine feed ad libitum and provided water ad libitum via an automatic watering system. Containment units were cleaned daily. Animals were monitored twice daily (morning and afternoon) for overall health, feed/water intake, and clinical signs. Rectal temperatures were recorded daily.

Pre-vaccination blood samples collected on Day 0 revealed the presence of PCV and PRRSV in some pigs, indicated by high Ct values (Table S3), suggesting a very low viral load at the start of the study.

### 2.3. Virus Inoculation and Challenge Study

**Figure 1:**
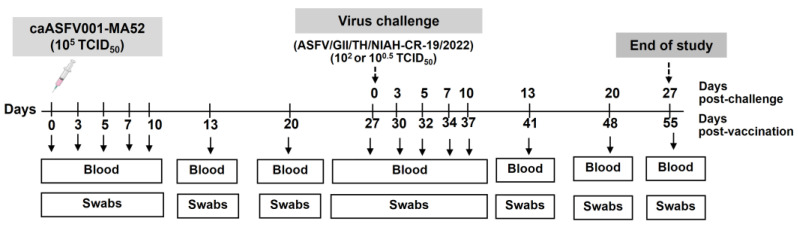
Experimental plan for inoculation with caASFV001-MA52. Pigs were given different caASFV001-MA52 doses on day 0, followed by challenge with the virulent ASFV strain ASFV/GII/Thailand/NIAH-CR-19/2022 at day 27 post-vaccination. Blood and swabs were collected at the various indicated time points.

Twenty-three pigs were randomly assigned to five experimental groups. Control groups A, B, and C were intramuscularly injected with PBS. Vaccination groups D and E were intramuscularly injected with 10^5^ TCID_50_ of caASFV001-MA52 per pig and challenged with virulent ASFV at either 10^2^ or 10^0.5^ TCID_50_ (Table 1). Following immunization, safety was assessed based on daily observations and measurements as previously described [25] that included recording rectal temperature, behavior (recumbency), anorexia, coughing, nasal and ocular discharges, skin, and gastrointestinal signs. Antibody and T cell responses were evaluated from blood samples. Viral DNA in blood, nasal, and oral swabs at the indicated time points was measured using qPCR (Figure 1).

After 27 days, vaccinated pigs were inoculated with ASFV/GII/Thailand/NIAH-CR-19/2022 at a dose of 10^2^ TCID_50_ (groups B and D) or 10^0.5^ TCID_50_ (groups C and E). After exposure, body temperature and clinical symptoms were monitored, and samples were collected at indicated time points (Figure 1). Animals were evaluated using predetermined endpoint criteria, which detail how individual parameters are scored from 0 (normal) to 3 (severe) to achieve a total cumulative clinical score. Animals were euthanized immediately if their total clinical score reached 10 or more or upon meeting specific qualitative criteria [25]. Spontaneous deaths were recorded as non-survivors.

At the end of the study, tissue samples were collected (tonsils, lung, spleen, kidney, liver, salivary gland, and lymph nodes) for qPCR quantification of viral load and histopathologic analysis.

### 2.4. Quantitative PCR (qPCR) Analysis

Total DNA was extracted using the DNeasy Blood & Tissue Kit (Qiagen, Hilden, Germany) according to the manufacturer’s instructions. ASFV genomic DNA was quantified via qPCR, using primers and a probe targeting the MGF360-21R gene. qPCR reactions were prepared in 20 µL containing 1× Luna Universal Probe qPCR Master Mix (New England Biolabs, Ipswich, MA, USA), 0.4 µM of each primer (forward: 5′-GGCTATCCTCATCAATGCAAATC-3′; reverse: 5′-GGCCACACAGCACATATCTA-3′), 0.2 µM of probe (5′-FAM-TATGGTGGCATGAAGCGCCGATTA-3BHQ-3′), and 2 µL of DNA sample. Amplification conditions included initial denaturation at 95 °C for 1 min, followed by 45 cycles of denaturation at 95 °C for 15 s and extension at 60 °C for 30 s, with fluorescence detection in the FAM channel at the end of each extension step.

A standard curve was generated using 10-fold serial dilutions of a plasmid encoding MGF360-21R from 10^9^ to 10^2^ copies. The standard curve was determined by plotting the log of target concentration against Ct values. qPCR results were expressed as log_10_ ASFV copies/mL (for serum samples) or log_10_ ASFV copies/gram (for tissue samples), with a Ct threshold under 40 cycles defining positivity. The lowest detection limit for the assay is 1000 copies/mL. All qPCR reactions were performed on a CFX Opus 96 Real-Time PCR System (Bio-Rad Laboratories, Hercules, CA, USA).

### 2.5. Enzyme-Linked Immunospot (ELISpot) Assay

To assess ASFV-specific T-cell responses, 10 mL of blood was collected from each pig. PBMCs were isolated by diluting blood 1:1 with PBS containing 2% FBS, followed by layering in SepMate™-50 tubes over Lymphoprep™ (StemCell Technologies, Vancouver, BC, Canada) and centrifugation at 1200× *g* for 10 min to separate PBMCs from red blood cells. PBMCs were washed twice with PBS containing 2% FBS, resuspended, and seeded into ELISPOT plates pre-coated with anti-porcine IFN-γ (ImmunoSpot^®^, Cellular Technology Limited, Rutesheim, Germany) at a density of 5 × 10^5^ cells/well. Cells were stimulated with ASFV at a multiplicity of infection (MOI) of 0.1 for 24 h. Plates were developed according to the manufacturer’s protocol. Briefly, the plate was washed four times (twice with PBS, twice with 0.05% Tween-PBS), followed by the addition of anti-porcine IFN-γ (Biotin) detection solution. After incubation for 2 h at room temperature, the plate was washed with 0.05% Tween-PBS followed by tertiary solution (Strep-AP) and incubated for 30 min. After the final wash with 0.05% Tween-PBS and distilled water, blue developer was added for 15 min to initiate the color reaction. Spots corresponding to ASFV-specific IFN-γ-secreting cells were counted using an S6 Micro M2 ELISpot Reader (ImmunoSpot^®^, Cellular Technology Limited).

### 2.6. Enzyme-Linked Immunosorbent Assay (ELISA)

ASFV-specific IgG antibodies were measured using the ID Screen^®^ ELISA kit (Innovative Diagnostics), following the manufacturer’s instructions. Pre-antigen-coated ELISA plates were incubated with 1:20 diluted serum samples, alongside positive and negative controls, for 45 min at room temperature (RT). Wells were then washed three times with 1× wash buffer. After washing, 1× conjugated detection antibody was added to each well, followed by incubation at RT for 30 min. After washing, substrate solution was added and incubated for 15 min at RT in the dark for signal development. The reaction was stopped with stop solution, and absorbance was measured at 450 nm using an EnSight™ multimode plate reader (PerkinElmer, Waltham, MA, USA). Data were analyzed using Kaleido™ software v3.5 (PerkinElmer).

### 2.7. Histopathology

Formalin-fixed tissues were processed for histological analysis following standard protocols for histopathological examination. Tissue sections were paraffin-embedded and stained with hematoxylin and eosin (H&E) to assess pathological changes. Histopathological lesions were systematically scored based on hemorrhage, tissue necrosis, degeneration, and inflammation. Lesion severity was classified into four categories: no remarkable lesions present (0), focal or multifocal lesions affecting ≤10% of the total tissue area (1), multifocal to diffuse lesions covering 10–50% of the total tissue area (2), and extensive lesions affecting >50% of the total tissue area (3).

### 2.8. Statistical Analysis

The Shapiro–Wilk test was used to assess whether the data met the assumptions for parametric analysis. Viral DNA levels in various samples and histological scores were Log10 transformed to achieve a normal distribution. The data that met the assumptions were assessed using a two-way analysis of variance (ANOVA) followed by Tukey’s multiple comparisons test to evaluate differences between treatment groups. Comparison of survival curves was determined using the Mantel–Cox log-rank test. Results are expressed as mean ± standard deviation (SD) unless stated otherwise, with a significance threshold set at *p* < 0.05. All statistical tests were conducted with 95% confidence intervals (CI). All data were analyzed using GraphPad Prism v9.0.0.

## 3. Results

### 3.1. Genomic Analysis of caASFV001-MA52

**Figure 2:**
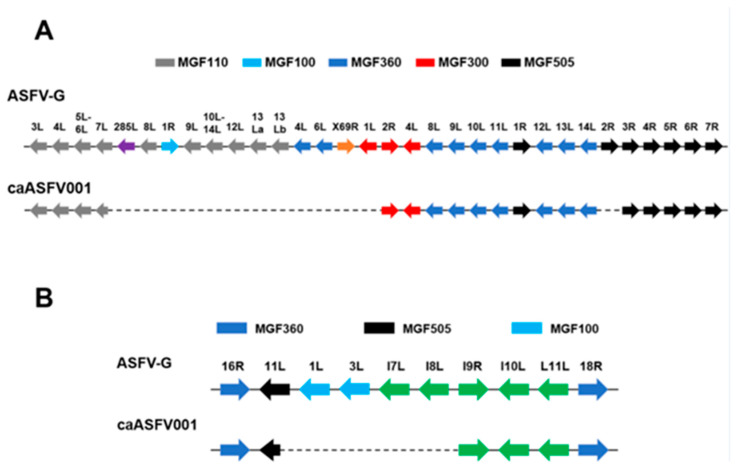
Schematic representations of the observed deletions in the caASFV001-MA52 genome. The caASFV001-MA52 genome’s (**A**) left variable region (LVR) and (**B**) right variable region (RVR) were aligned with respect to the ASFV Georgia 2007/1 (ASFV-G) sequence. Dashed lines indicate deleted regions within the viral genome. Multigene family (MGF) and non-MGF genes are color-coded. Non-MGF genes are omitted for clarity.

The genome of caASFV001-MA52 was sequenced and analyzed in comparison to the reference Georgia 2007/1 strain (GenBank accession number NC_044959.2). A major 11,018 bp deletion was identified in the left variable region (LVR), encompassing 23 MGF and non-MGF genes from the truncated N-terminal fragment of MGF110-7L to the deleted MGF300-1L gene (Figure 2A). Additionally, a 1088 bp deletion removed the central fragment of MGF505-2R, likely rendering this gene non-functional (Figure 2A). The right variable region (RVR) exhibited a 3458 bp deletion, affecting five genes, spanning from the truncated N-terminal fragment of MGF505-11L to the deleted I8L gene (Figure 2B). In total, 22 genes were deleted and 2 genes were truncated in the LVR [MGF110-7L (truncated), 285L, ACD_00160, MGF110-8L, MGF100-1R, ACD_00190, MGF110-9L, ACD_00210, MGF110-10L-MGF110-14L fusion, ACD_00240, MGF110-12L, MGF110-13La, MGF110-13Lb, ACD_00270, MGF360-4L, ACD_00300, MGF360-6L, ACD_00320, ACD_00330, ACD_00350, ACD_00360, X69R, MGF300-1L, and MGF505-2R (truncated)], while 4 genes were deleted and 1 gene truncated in the RVR [MGF505-11L (truncated), MGF100-1L, MGF100-3L, I7L, and I8L].

Deletion of key gene families, including MGF110, MGF360, and MGF505, may play a role in attenuation, as deletion of these genes has been correlated with reduced pathogenicity [26,27,28]. For example, some LAV candidates with engineered deletion of these genes, such as ASFV-G-ΔMGF, demonstrated reduced virulence combined with induction of protective immune responses in pigs [12,29]. A particular deletion in caASFV001-MA52 is the immune regulator gene MGF505-2R, which is involved in macrophage modulation, control of interferon responses, and viral attenuation [29,30,31,32]. Strains of ASFV carrying deletions in MGF505 have proven to be attenuated while remaining immunogenic; we hypothesize that these genomic deletions attenuate the caASFV001-MA52’s immunosuppressive potential.

### 3.2. Immunization with caASFV001-MA52

Pigs were divided into five experimental groups (Table 1) and inoculated with either PBS or caASFV001-MA52 at 10^5^ TCID_50_. The vaccine dose was selected based on pilot studies that defined the optimal balance between protection and safety. Preliminary data demonstrated that a dose of 10^6^ TCID_50_ achieved complete protection against a lethal challenge. Following immunization at this dose, viral replication was transient, peaking at 10^4^ copies/mL on day 5 post-vaccination and clearing completely by day 10, with no adverse clinical effects observed (Figures S1–S3). Conversely, a higher dose of 10^7^ TCID_50_ resulted in a less favorable safety profile, presenting a higher viral load (peak at 10^7^ copies/mL) and sustained viral presence up to day 21. It also resulted in one pig succumbing to septicemia co-infection with *S. suis* serotype 8 (Figures S3 and S4). This mortality was associated with severe inflammation and congestion in several organs (Figure S4). Based on these preliminary results, the 10^5^ TCID_50_ dose was chosen for the present study to specifically test efficacy and safety at a lower dose, specifically within the challenging context of a co-infected pig population.

#### 3.2.1. Clinical Findings Post-Vaccination

**Figure 3:**
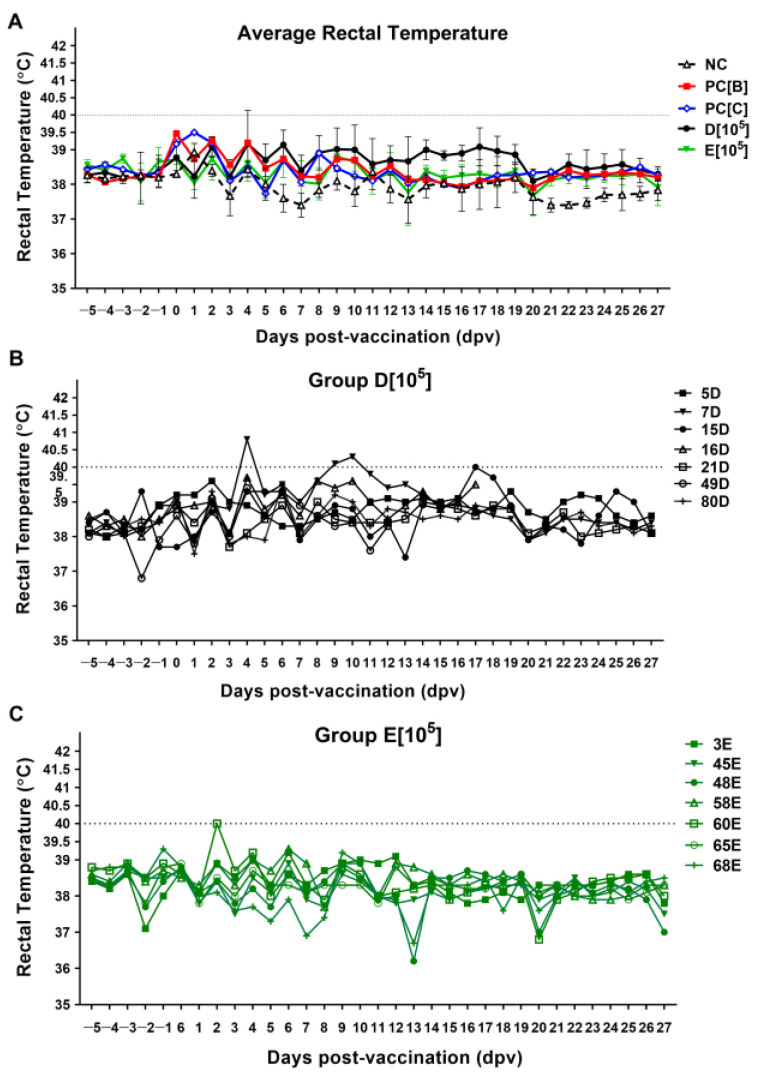
The temperature of pigs in each group post-vaccination. (**A**) Rectal temperatures were measured daily and calculated as the average rectal temperature of pigs in each group; error bars represent mean ± SD, or individual pig values are shown for experimental groups (**B**) D[10^5^] and (**C**) E[10^5^]. Negative control (NC), PC[B], and PC[C] received PBS. Group D[10^5^] and E[10^5^] received caASFV001-MA52 at a dose of 10^5^ TCID_50_.

After vaccination, rectal temperatures were recorded daily during the entire trial, while fever and general health were evaluated according to the following criteria [25]. Most of the vaccinated pigs maintained a rectal temperature within the normal range (37.0–39.5 °C) for the 27 days before the challenge (Figure 3A). A transient elevation in mean body temperature was observed in group D[10^5^] during the first and second weeks post-vaccination (Figure 3A). While the group mean remained below the fever threshold, temperatures in this group ranged from 39.5 to 40.5 °C during this period (Figure 3B). This temperature elevation was clinically self-limiting, and the pigs continued to consume feed and water normally without exhibiting distinct clinical signs of ASF.

A transient, self-limiting diarrhea was observed in five pigs (66C, 5D, 49D, 45E, 68E) between 6 and 8 days post-vaccination (dpv). These pigs maintained normal food consumption, water intake, and body temperature. Subsequent qPCR fecal swab screening confirmed the presence of *S. suis* in pigs 5D and 49D, and PCV2/PCV3 in pigs 5D and 45E (Table S4). Pig 49D further developed lameness and articular swelling. This pig succumbed to death at 13 dpv. Necropsy showed severe pneumonia and petechial hemorrhage in the tonsil and kidney, and splenomegaly with infarction (Figure S5). PCR screening of synovial fluid confirmed the presence of ASFV and *S. suis*, suggesting potential co-infection and immunosuppression. Another fatal case after vaccination was observed in pig 16D on day 18 dpv. Necropsy indicated severe pneumonia and petechial hemorrhage in the tonsil and kidneys, and splenomegaly with infarction. Crucially, the gut and inguinal lymph nodes showed no significant inflammation (Figure S5).

The overall investigation, confirmed by necropsy and PCR, concluded that underlying bacterial infection, particularly *S. suis*, was involved in the mortality of these pigs. While the exact effect of caASFV001-MA52 on disease severity remains uncertain, poor host health and latent infections are hypothesized to have exacerbated ASFV replication, resulting in lethal outcomes. The rapid and peracute disease progression, particularly in the presence of co-infection, is hypothesized to have resulted in the animals succumbing spontaneously before exhibiting the cumulative severe clinical signs necessary to trigger the humane endpoint. Major clinical signs in some pigs’ post-vaccination were summarized in Table 2.

The pre-challenge mortality observed in group D[10^5^] was not consistent across all vaccinated cohorts, mitigating the possibility of sole vaccine residual virulence. Specifically, group E[10^5^] that received caASFV001-MA52 at the identical dose, yet experienced zero pre-challenge mortality and remained in good clinical condition. This key finding is supported by our preliminary study (Figure S1), where pigs from a different source herd, confirmed negative for all pre-existing viral and bacterial co-infections, exhibited no adverse reactions even when administered a higher dose (10^6^ TCID_50_) of the same caASFV001-MA52 batch. These collective results provide evidence that the pre-challenge mortality observed in group D[10^5^] was most likely due to the exacerbation of latent co-infections.

#### 3.2.2. caASFV001-MA52 Detection and Pathogenic Viral and Bacterial Reactivation Post-Vaccination

**Figure 4:**
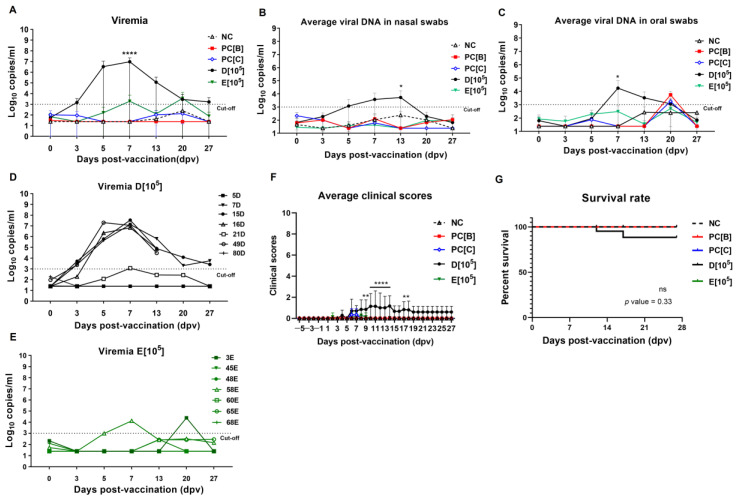
ASFV DNA levels in samples, clinical score, and survival post-vaccination. (**A**) Average viral DNA in (**A**) blood, (**B**) nasal, and (**C**) oral swab samples at various time points was analyzed by qPCR. Data points represent the mean virus titer (expressed in Log_10_ copies/mL) ± standard deviation (SD). (**D**,**E**) viral DNA in blood samples of individual pigs in groups D[10^5^] and E[10^5^]. (**F**) Average daily cumulative clinical scores (CCS). Data points represent the mean daily CCS ± SD. The significance between group D[10^5^] and E[10^5^] was calculated using two-way ANOVA and Tukey’s multiple comparison test. **** *p* < 0.0001, ** *p* < 0.01, * *p* < 0.05. (**G**) Percent survival in each group post-vaccination (pre-challenge). A survival curve was calculated for the various experimental groups using the log-rank (Mantel–Cox) test. ns, no significant difference. Negative control (NC), PC[B], and PC[C] received PBS saline. Group D[10^5^] and E[10^5^] received caASFV001-MA52 at a dose of 10^5^ TCID_50_.

ASFV viral load was at varying levels and different between the vaccinated groups (Figure 4A). Pigs in group D[10^5^] had the highest viremia at 7 dpv (mean ± SD: 9.44 × 10^6^ ± 1.25 × 10^7^ copies/mL), which likely correlated with their health condition. Viral DNA in blood and swabs in most pigs appeared significantly at 7 to 13 dpv (Figure 4B,C). Pigs 49D and 16D that succumbed in the study showed high viral loads, probably exacerbated by bacterial co-infection. The remaining pigs had very low viral viremia (mean ± SD: 1.65 × 10^3^ ± 2.47 × 10^3^ copies/mL) by 27 dpv (Figure 4D).

In group E[10^5^], the majority of blood and swab samples tested negative, excluding pigs 58E and 3E. Viremia in this group was lower (mean ± SD: 1.92 × 10^3^ ± 5.01 × 10^3^ copies/mL) compared with group D[10^5^] at 7 dpv (Figure 4A–C). Individual viral DNA in blood, nasal, and oral swabs is shown in Figures S6–S8. In summary, vaccinated pigs in group D[10^5^] had a higher clinical score and lower survival rate (mean ± SD: 1.16 ± 0.98 and 71.4%, respectively) compared with those in group E[10^5^] (mean ± SD: 0.28 ± 0.48 and 100%, respectively) (Figure 4E,F). This highlights the critical challenge of residual virulence susceptibility in LAVs when tested in a co-infected pig population.

Blood samples were also evaluated for the presence of other pathogens at different time points post-vaccination. We found that some vaccinated pigs (21–35% of the total number of vaccinated pigs) were PCV2- and PCV3-positive (Table 3), with Ct values ranging from 36 to 39, indicating low viral load during the first week of vaccination (Table 3 and Table S4). PRRSV (both EU- and US-strain) remained detectable in 14% of vaccinated pigs at 7 to 27 dpv (Table 3). However, PCV2 and PCV3 were eliminated from all vaccinated animals by 13 dpv (Tables S5–S7). This differential outcome suggests that while the vaccine or co-infection may have transiently supported early virus replication, the host immune response was effective in clearing PCV2 and PCV3 following peak replication. No bacteremia was detected, although *S. suis* was found in the feces of some diarrheic pigs (Table 2).

### 3.3. ASFV Challenge and Clinical Outcomes

**Figure 5:**
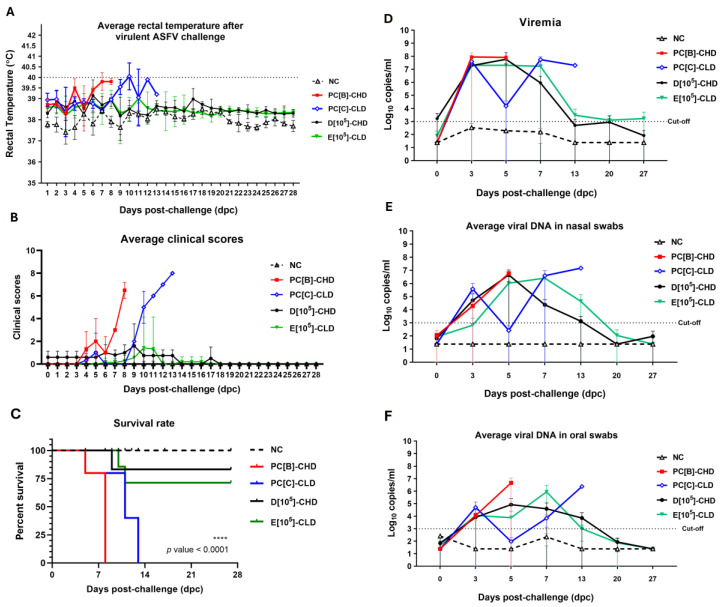
Average rectal temperature, clinical scores, survival, and viral load in the samples of challenged pigs. (**A**) Body temperatures were measured at the indicated time points. Data points represent the mean ± SEM. (**B**) Daily average cumulative clinical scores. Data points represent the mean ± SD. (**C**) A survival curve was calculated for the various experimental groups using the log-rank (Mantel–Cox) test. ASFV DNA copy number was quantified in (**D**) blood samples, (**E**) nasal swabs, and (**F**) oral swabs by qPCR. Data points represent the mean ± SD. Negative control or NC, PC[B]-CHD, and PC[C]-CLD were given PBS. NC was not challenged, while PC[B]-CHD and PC[C]-CLD were challenged at a dose of 10^2^ and 10^0.5^ TCID_50_, respectively. Group D[10^5^]-CHD received 10^5^ TCID_50_ of caASFV001-MA52 and 10^2^ TCID_50_ of the challenge virus. Group E[10^5^]-CLD received 10^5^ TCID_50_ of caASFV001-MA52 and 10^0.5^ TCID_50_ of the challenge virus.

Pigs were intramuscularly inoculated with the challenge virus using different doses; note that only five of seven pigs in group D[10^5^] remained available for the challenge (Table 1). Control groups PC[B]-CHD and PC[C]-CLD, which were challenged with 10^2^ and 10^0.5^ TCID_50_ of virulent ASFV, experienced disease progression with body temperatures increasing to 40 °C by 7–11 days post-challenge (dpc) (Figure 5A). Not all pigs survived, with one humanely euthanized and two succumbing spontaneously by 8 dpc, and two humanely euthanized and one succumbing spontaneously by 13 dpc, respectively (Figure 5B). These results confirm the 100% lethality of the virulent ASFV strain used in the challenge (Figure 5C). Viral DNA was detected in blood, nasal, and oral samples of both groups in the week following the challenge (Figure 5D–F; individual plots in Figures S6–S8).

In the vaccinated groups, D[10^5^]-CHD and E[10^5^]-CLD, pigs largely maintained body temperatures below 39.5 °C (Figure 5A; individual plots in Figure S9). Pig 80D presented mild fever and reduced intake before succumbing on 10 dpc. In group E[10^5^]-CLD, all pigs were mildly febrile (39.5–40.2 °C) during the challenge. Survival rate was 80% in group D[10^5^]-CHD and 71.4% in group E[10^5^]-CLD (Figure 5B,C) with similar average clinical scores (D[10^5^]-CHD: 1.60 ± 1.94; E[10^5^]-CLD: 1.42 ± 2.69). The surviving pigs presented stable temperatures and low clinical scores (individual clinical score plot, Figure S10). Furthermore, the viral load, which had initially increased during the first week post-challenge, dropped significantly during the second week in both blood and swabs (Figure 5D–F; individual plots in Figures S6–S8). These findings suggest that caASFV001-MA52 at 10^5^ TCID_50_ could confer protection, resulting in a 70–80% reduction in mortality, viral load, and disease severity against the virulent ASFV challenge.

### 3.4. Immune Responses of Vaccinated Pigs

**Figure 6:**
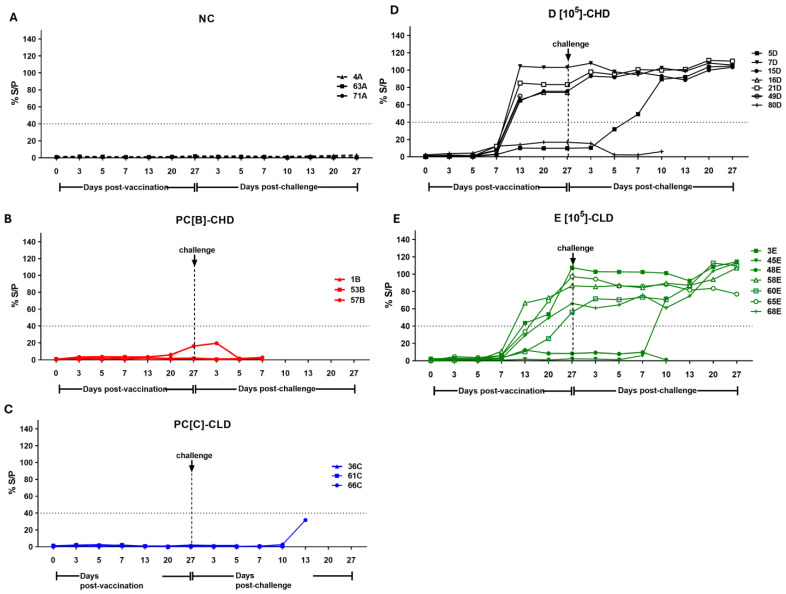
Antibody response in individual pigs before and after the challenge. Blood samples were collected at the indicated time points. Antibody levels were measured using the ID Screen^®^ African Swine Fever Indirect ELISA kit. The sample to positive (S/P) ratio was calculated for each sample (dotted lines, cut-off = 40% S/P). (**A**) Negative control or NC, (**B**) PC[B]-CHD, and (**C**) PC[C]-CLD were given PBS. NC was not challenged, while PC[B]-CHD and PC[C]-CLD were challenged at a dose of 10^2^ and 10^0.5^ TCID_50_, respectively. (**D**) Group D[10^5^]-CHD received 10^5^ TCID_50_ of caASFV001-MA52 and 10^2^ TCID_50_ of the challenge virus. (**E**) Group E[10^5^]-CLD received 10^5^ TCID_50_ of caASFV001-MA52 and 10^0.5^ TCID_50_ of the challenge virus.

**Figure 7:**
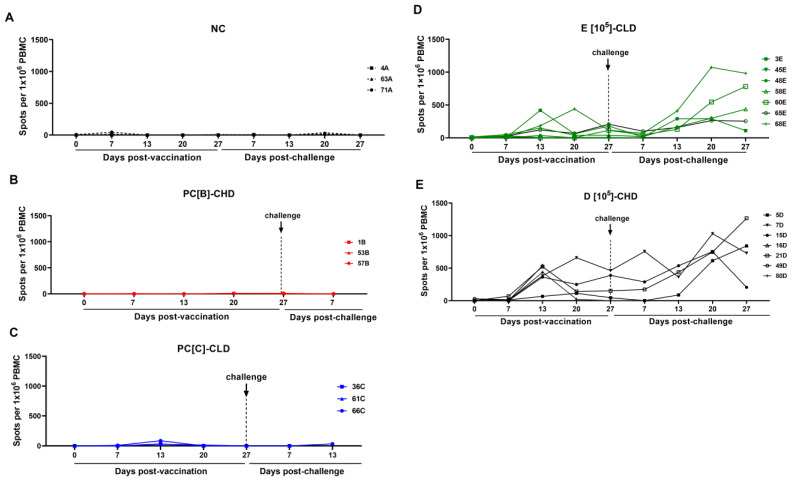
T-cell response in individual pigs before and after the challenge. PBMCs were harvested at the indicated time points and stimulated with ASFV at an MOI of 0.1. Specific anti-ASFV responses were measured by counting the number of spots on the membrane of the porcine IFN-γ ELISpot assay. Results are expressed as spots per 10^6^ PBMCs. (**A**) Negative control or NC, (**B**) PC[B]-CHD, and (**C**) PC[C]-CLD were given PBS. NC was not challenged, while PC[B]-CHD and PC[C]-CLD were challenged at a dose of 10^2^ and 10^0.5^ TCID_50_, respectively. (**D**) Group D[10^5^]-CHD received 10^5^ TCID_50_ of caASFV001-MA52 and 10^2^ TCID_50_ of the challenge virus. (**E**) Group E[10^5^]-CLD received 10^5^ TCID_50_ of caASFV001-MA52 and 10^0.5^ TCID_50_ of the challenge virus.

Looking at the immune responses to ASFV post-vaccination and post-challenge, none of the control groups seroconverted, and no detectable T-cell responses were observed (Figure 6A–C and Figure 7A–C), reflecting the 0% survival rate. Within group D[10^5^]-CHD, most pigs (7D, 15D, and 21D) had high antibody levels (%S/P ratio: 80–110%) and T-cell responses (500–600 spots/10^6^ PBMC) pre-challenge, which were significantly increased by 20–27 dpc, and survived the ASFV challenge. The initially low antibody responder (pig 5D) showed a strong immune response after the challenge and survived. In contrast, pig 80D had consistently low antibody and T-cell responses and failed to acquire efficient immunity (Figure 6D and Figure 7D). Group E[10^5^]-CLD had lower pre-challenge immune responses than group D[10^5^]-CHD; while some pigs recovered from infection, others did not mount an adequate immune response for protection (45E and 48E) (Figure 6E and Figure 7E).

### 3.5. Disease Development and Lesion After Virulent ASFV Infection

**Figure 8:**
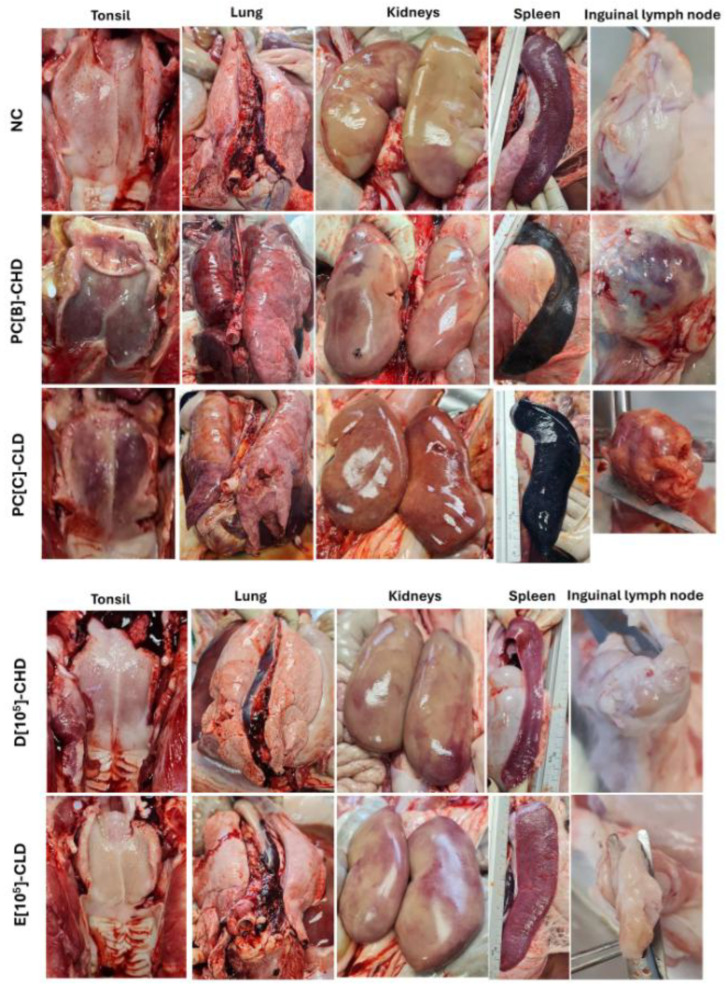
Gross pathological findings at necropsy. Upon necropsy after euthanasia at humane endpoints or at the end of the trial, gross pathology was examined in various organs of pigs in each group. Representative images of tonsils, lungs, kidneys, spleens, and inguinal lymph nodes are shown. Negative control or NC, PC[B]-CHD, and PC[C]-CLD were given PBS. NC was not challenged, while PC[B]-CHD and PC[C]-CLD were challenged at a dose of 10^2^ and 10^0.5^ TCID_50_, respectively. Group D[10^5^]-CHD received 10^5^ TCID_50_ of caASFV001-MA52 and 10^2^ TCID_50_ of the challenge virus. Group E[10^5^]-CLD received 10^5^ TCID_50_ of caASFV001-MA52 and 10^0.5^ TCID_50_ of the challenge virus.

At the end of the experiment, all survivors were humanely euthanized for gross pathological observation, viral load in tissues, and histopathology. Necropsy revealed normal internal organs in the negative control (NC), whereas severe ASF-associated hemorrhage and congestion were found throughout the visceral organs of the positive control groups PC[B]-CHD and PC[C]-CLD (Figure 8). Survivors in group D[10^5^]-CHD (5D and 7D) and E[10^5^]-CLD (60E and 65E) exhibited well-preserved tissue structure without evidence of hemorrhage, congestion, or necrosis in vital organs, similar to the negative control (Figure 8). The presence of focal splenic infarcts (often at the tip of the spleen) and mild splenic congestion was found in some pigs (Figure S11), indicating non-lethal vascular damage associated with subclinical ASFV challenge.

**Figure 9:**
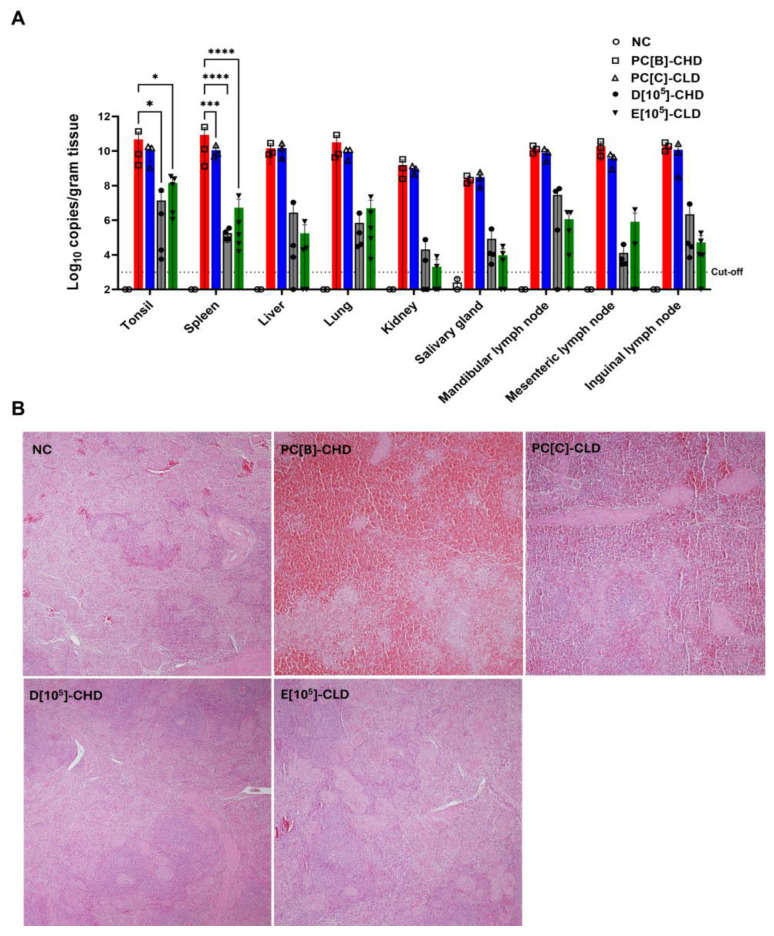
Viral DNA in various tissues and histopathological findings of spleen samples of control pigs and surviving vaccinated pigs. (**A**) Various tissues were collected and subjected to qPCR to determine viral DNA. Data points represent the mean (expressed in Log_10_ copies/gram) ± SD. A two-way analysis of variance (ANOVA) followed by Tukey’s multiple comparisons test was performed to evaluate differences between treatment groups. * *p* < 0.05, *** *p* < 0.001, **** *p* < 0.0001. (**B**) Histopathological evaluation. Tissues (spleen as a representative) were stained with hematoxylin and eosin. The pictures were taken at the original magnification of 20×. The NC group showed no evidence of tissue necrosis or inflammatory cell infiltration. In contrast, the PC[B]-CHD group exhibited severe acute diffuse necrohemorrhage of the lymphoid tissue with complete disruption of the splenic microstructure and absence of white pulp. The PC[C]-CLD group demonstrated a moderate decrease in the number of white pulps accompanied by moderate multifocal lymphoid necrosis, moderate congestion of the red pulp, and mild hemorrhage. In group D[10^5^]-CHD, the white pulp showed a moderate reduction in size and number with mild to moderate lymphoid necrosis; congestion of the red pulp was absent, although mild hemorrhage was observed. The E[10^5^]-CLD group revealed a mild to moderate reduction in the size and number of white pulps with mild lymphoid necrosis and no evidence of congestion in the red pulp. Negative control or NC, PC[B]-CHD, and PC[C]-CLD were given PBS. NC was not challenged, while PC[B]-CHD and PC[C]-CLD were challenged at a dose of 10^2^ and 10^0.5^ TCID_50_, respectively. Group D[10^5^]-CHD received 10^5^ TCID_50_ of caASFV001-MA52 and 10^2^ TCID_50_ of the challenge virus. Group E[10^5^]-CLD received 10^5^ TCID_50_ of caASFV001-MA52 and 10^0.5^ TCID_50_ of the challenge virus.

The viral DNA of survivors remained in various tissues, however, at lower levels than in the positive controls (Figure 9A). Histopathological analysis of the spleen supported the gross pathological findings and confirmed the protective efficacy of caASFV001-MA52. In the positive control groups, PC[B]-CHD and PC[C]-CLD, spleens exhibited complete effacement of normal lymphoid architecture, severe lymphocyte depletion in the white pulp, and extensive hemorrhage and necrosis throughout the red pulp (Figure 9B). Therefore, pigs in this group exhibited severe immunosuppression accompanied by vasculitis and vascular leakage in multiple organs. By contrast, in the vaccinated groups D[10^5^]-CHD and E[10^5^]-CLD, the splenic architecture was well-preserved or only mildly disrupted, maintaining a clear distinction between red and white pulp, evidence of lymphoid hyperplasia, and marked reductions in hemorrhages (Figure 9B and Figure S12). Hemorrhage and necrosis were either absent or highly localized to small, focal areas of infarction, supporting the observation that the vaccine limited viral pathogenesis to subclinical levels.

## 4. Discussion

ASFV is known to possess highly advanced immune evasion strategies that enable it to regulate macrophage activities, inhibit IFN signaling, and manipulate antigen processing and presentation [33,34,35]. These immunosuppressive strategies serve as major barriers to vaccine development. Consequently, effective live vaccines must be optimally attenuated while inducing strong anti-viral immune responses. Serial passage of ASFV in cell culture is a viable strategy for viral attenuation. Other cell-adapted strains have demonstrated replication, low virulence, and immunogenicity [36].

Genomic deletions in caASFV001-M52, particularly in multigene family (MGF) regions, likely impair the ability of the virus to evade the host immune system [35]. Deletion of MGF110, MGF360, and MGF505 have been linked with reduced pathogenicity [26,27,28]. The immune regulator gene MGF505-2R is involved in macrophage modulation and interferon control, and its deletion also contributes to attenuation [29,30,31,32]. Our data demonstrates that caASFV001-MA52 stimulates a strong immune response, decreases viral load, and increases survival in ASFV-challenged pigs. Protection was dose-dependent, with 100% protection at the highest dose (Figure S1). The observation of variable immune responses across the vaccinated groups, with some animals showing strong antibody and T cell responses and others showing low or no response, is an expected phenomenon in an outbred, commercial pig population. This biological heterogeneity is likely driven by genetic polymorphism in immune factors. Furthermore, the presence of latent or subclinical endemic co-infections such as *S. suis* and PCV2/3 likely resulted in immunological interference.

The 70–80% protection rate observed for caASFV001-MA52 at 10^5^ TCID_50_ is lower than the 100% protection rate reported for the other candidates, such as gene-deleted candidates (ASFV-G-∆I177L and ASFV-G-∆I177L∆LVR) [11,37] and the cell-adapted ASFVs (ASFV-MEC-01 and VNUA-ASFV-LAVL2) [20,38]. However, direct comparison is complicated by differences in the study design, vaccine doses, and challenge strain virulence. Most candidates reporting 100% efficacy were conducted in SPF pigs or clean herds, utilizing doses ranging from 10^2^–10^6^ HAD_50_ without observing post-vaccination adverse effects in that controlled environment. Conversely, our study evaluated safety and efficacy in pigs with pre-existing co-infections, a scenario not frequently reported or explored in detail in other studies. The transient virulence and fatalities we observed highlight a critical distinction regarding safety and efficacy under real-world field conditions. The protection rate may represent a necessary trade-off compared to the 100% protection seen in disease-free animals, as it reflects the performance of the LAV when its safety is being compromised by underlying conditions. This sensitivity is likely not unique to our candidate, as other LAV candidates, such as the VaCln3 P13 candidate (derived from a Korean ASFV isolate adapted to Vero cells), have similarly shown mortality driven by common secondary *S. suis* infection in the high-dose group [21]. This supports that LAVs, regardless of their specific genomic deletions or adaptation strategy, can impose a transient vulnerability to common co-pathogens in the field.

This study emphasizes the complications of using LAVs in pigs with pre-existing viral and bacterial co-infections. The pigs used in this study came from a commercial farm with latent PCV2, PCV3, and *Mycoplasma* spp., reflecting a real-world farm environment. The temporary reactivation of latent viruses and subsequent mortality from ASFV and S. suis co-infection strongly suggest that the caASFV001-MA52 candidate imposes a transient period of host immunosuppression. It is a significant safety concern, especially in animals that are already facing compromised health. These findings are consistent with the reported ASFV pathogenic mechanism of stimulating CD14-dependent phagocytosis by macrophages, which was associated with increased internalization of bacteria in co-infected animals and consequent amplification of inflammatory responses and enhancement of viral transmission in apoptotic bodies [39]. However, since other pigs given the same vaccine dose remained healthy, the mortality was likely caused by co-infection. This suggests that the interplay between immunosuppression and co-infections can compromise vaccine safety [21]. There may be a threshold or a certain mixture of co-infections that can cause the host system to be overstressed, resulting in vaccine failure or adverse events in some individuals.

The observed residual virulence and potential for transient immunosuppression in caASFV001-MA52 may be caused by the retention of key virulence determinants. Specifically, the retained MGF300-2R and MGF300-4L protein likely function to inhibit the NF-κB pathway by promoting the autophagic degradation of IKK components and stabilizing its inhibitor IκBα [40,41,42]. Furthermore, the retained MGF360-9L, MGF360-10L, and MGF360-14L genes may form a defense complex against the host antiviral response through their actions, which include disrupting interferon signaling, targeting IRF3 degradation, and antagonizing the JAK/STAT signaling pathway [43,44,45,46]. Given that the ASFV genome encodes hundreds of proteins, deleting every immunosuppressive protein is not feasible, as such over-attenuation would likely result in the virus being non-replicative or non-immunogenic, leading to vaccine failure [47]. We hypothesize that the cumulative effect of these retained virulent proteins ensures sufficient, prolonged viral replication in macrophages that is critical for generating durable protective immunity but may carry the inherent risk of transiently debilitating the host’s ability to control concurrent viral and bacterial infections. Finally, while NGS confirmed that the major population successfully mapped to the prototype genome featuring three large deletions, the possibility of a viral quasi-species retaining minor deletion cannot be ruled out, even after purification and sub-passaging. Consequently, to ensure prolonged safety, efficacy, and genetic stability of the seed virus, necessary quality control measures such as further safety tests, long-term monitoring, and rigorous genetic purity measurements must be continually applied.

We acknowledge that the vaccine dosage at 10^5^ TCID_50_ used in the study is substantially higher than some other LAV candidates and may lead to enhanced virulence, particularly during co-infection. This risk is magnified by the immunosuppressive mechanisms of common co-pathogens. PCV2 and PRRSV inhibit macrophage and dendritic cell activity, which is crucial for vaccine-induced immunity [48]. PCV2 enhances the expression of IL-10, an immunosuppressive cytokine that dampens immune activation [49]. A synergistic effect is plausible if the ASFV LAV by itself induces immune perturbation, which could reactivate or exacerbate these underlying infections. However, the major genomic deletions of MGF genes in our candidate likely provide partial attenuation against the wild-type virus’s immunosuppressive profile. We speculate that cell-adapted LAVs, which typically acquire large, random deletions during passage, may fundamentally require a higher effective dose compared with genetically engineered candidates that target specific virulent genes. For example, the ASFV-MEC-01 generated by serial passage of a field isolate in CA-CAS-01-A cells has an optimal dose up to 10^5^ HAD_50_ while maintaining a high level of protection and a good safety profile [20]. Future studies are needed to fine-tune the optimal effective dose and evaluate the immediate post-vaccination cytokine profile and macrophage functionality to confirm the degree and duration of residual immunosuppressive effects.

This study highlights how the efficacy and safety profiles of LAVs, such as caASFV001-MA52, can be affected by extrinsic conditions, particularly the physiological and environmental states of the vaccinated hosts. Stressors such as weaning, crowding, transport, and diet changes can impact an animal’s immune state. The observed fatalities (Pigs 49D and 16D) represented a peracute disease progression associated with co-pathogens confirmed at necropsy. Crucially, we speculate that caASFV001-MA52, despite being generally safe in healthy pigs, could produce harmful effects in such stressed animals through transient immune modulation, opening a window of susceptibility to other stressors or pathogens. To improve the practicality and safety of LAVs, comprehensive research is needed to understand ASFV and co-infections, ideally to identify specific co-pathogen combinations or host immune statuses that may carry the greatest risk of adverse effects. Research in immune modulation strategies, including nutrition-based supplements or targeted therapy, may be able to promote enhanced immunity during the temporary susceptibility following immunization. Safety trials under the challenging conditions found in actual farming environments need to be performed, as ‘proof-of-principle’ field trials in controlled environments.

An optimal ASFV LAV vaccination strategy requires high-quality farm and biosecurity management. Veterinary supervision is crucial in the form of pre-vaccine health screening focused on detecting infections and continued surveillance. Measures to reduce stress, such as stable social groups and proper nutrition, are also important. Timely diagnosis, prophylaxis, and treatment of co-infections, as well as proactive biosecurity measures, help create an environment where a vaccine can be most effective. Ultimately, a combined approach of an efficacious vaccine and intensive biosecurity is the most promising strategy for achieving sustainable ASF control.

## 5. Limitations of the Study

The protective efficacy and safety profile in co-infected pigs observed in our study utilized a small, genetically uniform, and controlled cohort. The results may represent the maximum achievable efficacy under ideal experimental conditions. The small sample size in this preliminary study limits the statistical power to detect rare adverse events or subtle differences in immune response. Future development must transition to larger-scale farm trials to precisely quantify the safety profile, confirm the immunological threshold for protection, and accurately determine the vaccine’s efficacy and impact on transmission dynamics under highly heterogeneous, real-world conditions of commercial pig production.

While the major genomic deletions of MGF genes in our candidate are presumed to have partially attenuated and enhanced virulence in co-infected pigs, a definitive evaluation of the vaccine’s residual immunosuppression is constrained. Specifically, because histopathological evaluation was performed only after the lethal challenge, it is difficult to isolate the pathology caused by the caASFV001-MA52 vaccine alone from that caused by the virulent challenge virus. Although findings from our safety test (at a dose of 10^6^ TCID_50_) did reveal a mild and transient decrease in white pulp in the spleens of some vaccinated pigs, suggesting subtle pathology, we unfortunately lack crucial comparative data from co-infected pigs that received the vaccine but were not subsequently challenged. This comparison would be highly valuable to evaluate whether the vaccine-induced transient immune perturbation, when combined with latent co-infections, enhances the severity of lymphoid lesions or exacerbates co-pathogen replication.

## 6. Conclusions and Future Perspectives

This study elucidates a vital interplay between the vaccine, pre-vaccination host status, and the farm environment that likely accounts for the adverse effects detected in some vaccinated pigs. A disease control approach encompassing complete farm management and rigorous biosecurity measures is paramount for the safe and effective application of caASFV001-MA52 and other LAVs in commercial conditions. Overall, caASFV001-MA52 represents an important step forward in ASF vaccine development, demonstrating significant immunogenicity and protective efficacy against ASFV challenge. For caASFV001-MA52 to be considered a viable commercial solution, future studies must focus on assessing long-term efficacy and safety, which includes conducting comprehensive testing to rule out the establishment of a carrier state by analyzing prolonged viral shedding in various tissues. By optimizing the design of field trials and investigating these immune–pathogen interactions, we expect this candidate to contribute meaningfully to controlling ASFV, reducing economic losses, and improving swine health in endemic areas.

## 7. Patent

A patent application related to the African swine fever vaccine candidate, caASFV001-MA52, has been filed by the National Science and Technology Development Agency.

## Figures and Tables

**Table 1:** Experimental groups.

Group	Inoculum	caASFV001-MA52 (TCID_50_)	No. of Pigs Pre-Vaccination	No. of Pigs Post-Vaccination	Challenge Virus (TCID_50_)	Abbreviation
A	PBS	-	3	3	-	NC
B	PBS	-	3	3	10^2^	PC[B]-CHD
C	PBS	-	3	3	10^0.5^	PC[C]-CLD
D	caASFV001-MA52	10^5^	7	5 *	10^2^	D[10^5^]-CHD
E	caASFV001-MA52	10^5^	7	7	10^0.5^	E[10^5^]-CLD

NC: negative control; CHD: challenge, high dose (10^2^ TCID_50_); CLD: challenge, low dose (10^0.5^ TCID_50_). * Vaccinated pigs remain after vaccination.

**Table 2:** Summary of clinical signs in pigs post vaccination (pre-challenge).

Group	Label	Clinical Signs (Score)	Days Post-Vaccination	Remark
PC[C]	66C	Diarrhea (1)	6–7	
D[10^5^]	5D	Diarrhea (1)	6–7	*S. suis* and PCV2 detected in feces
	7D	Fever (1)	4, 9, 11	Smaller size
		Anorexia (1)	7–14	
	15D	Fever (1)	17–18	Smaller size
		Anorexia (1)	7–14	
	16D	Anorexia (1)	7–14	Smaller size/ Non-survivor
	49D	Diarrhea (1)	6–7	Non-survivor *S. suis* and caASFV001-MA52 were detected in joint fluid
		Joint swelling (1)	7–10	
		Joint swelling (2)	11–12	
		Anorexia (1)	11–12	
E[10^5^]	45E	Diarrhea (1)	6–8	PCV2 detected in feces
	68E	Diarrhea (1)	6–8	

**Table 3:** Percentage of blood samples positive for pathogenic viruses in vaccinated pigs.

Pathogen	0 dpv (N = 14)	7 dpv (N = 14)	13 dpv (N = 14)	20 dpv (N = 14)	27 dpv (N = 12)
PCV2	0	35.7 (5/14)	0	0	0
PCV3	7 (1/14)	21.4 (3/14)	0	0	0
PRRSV (EU)	0	14.2 (2/14)	14.2 (2/14)	0	0
PRRSV (US)	0	14.2 (2/14)	0	14.2 (2/14)	16.6 (2/12)
PRRSV (HP)	0	0	0	0	0

dpv: days post-vaccination.

## Data Availability

Data will be made available on request.

## Supplementary Materials

The following supporting information can be downloaded at: https://www.mdpi.com/article/10.3390/vaccines13121189/s1. Figure S1: Preliminary challenge study of caASFV001-MA52 at a dose of 10^6^ TCID_50_. Figure S2: ASFV DNA levels in various samples of individual pig in the preliminary challenge study of caASFV001-MA52 at a dose of 10^6^ TCID_50_. Figure S3: Preliminary safety study of caASFV001-MA52 at a dose of 10^6^ TCID_50_ and 10^7^ TCID_50_. Figure S4: Gross pathological findings of vaccinated pig at necropsy. Figure S5: Gross pathological findings in pigs 49D and 16D. Figure S6: ASFV DNA levels in blood samples of individual pigs pre- and post-challenge. Figure S7: ASFV DNA levels in nasal swabs of individual pigs pre- and post-challenge. Figure S8: ASFV DNA levels in oral swabs of individual pigs pre- and post-challenge. Figure S9: Rectal temperatures of individual pigs pre- and post-challenge. Figure S10: Daily cumulative clinical scores of individual pigs after challenge. Figure S11: Gross pathological findings of surviving pigs at necropsy. Figure S12: Average total lesion scores of controls and surviving pigs at necropsy. Table S1: PCR screening of viral and bacterial pathogens in serum samples from pigs on the farm prior to entering the experimental facility (1st screening). Table S2: Anti-ASFV antibody levels in pig serum samples prior to entering the experimental facility. Tables S3–S7: PCR screening of viral and bacterial pathogens in serum samples of individual pigs (0–27 days post-vaccination).

## Abbreviations

The following abbreviations are used in this manuscript:

ASFV	African swine fever virus
caASFV	Cell-adapted African swine fever virus
CHD	Challenge, high dose
CLD	Challenge, low dose
CSFV	Classical swine fever virus
Ct	Threshold cycles
DMEM	Dulbecco’s Modified Eagle Medium
DPV	Days post-vaccination
DPC	Days post-challenge
ELISA	Enzyme-linked immunosorbent assay
ELISpot	Enzyme-linked immunosorbent spot
HAD_50_	Median hemadsorption dose
H&E	Hematoxylin and eosin
IFN-γ	Interferon gamma
LAV	Live attenuated vaccine
LVR	Left variable region
Mhp	*Mycoplasma hyopneumoniae*
Mhr	*Mycoplasma hyorhinitis*
MOI	Multiplicity of infection
NC	Negative control
PBMCs	Peripheral blood mononuclear cells
PBS	Phosphate-buffered saline
PCV	Porcine circovirus
PRRSV	Porcine reproductive and respiratory syndrome virus
RPMI-1640	Roswell Park Memorial Institute (RPMI) 1640 medium
RVR	Right variable region
qPCR	Quantitative polymerase chain reaction
SPF	Specific-pathogen-free (SPF)
*S. suis*	*Streptococcus suis*
TCID_50_	Median tissue culture infectious dose
