# Supplementary material for: Evaluation of a Cell-Adapted Live Attenuated African Swine Fever Virus Thai-Strain Vaccine Candidate: Highlighting Enhanced Virulence Risk in Co-Infected Pigs
Source: Vaccines (Basel). 2025 Nov 24;13(12):1189. doi: 10.3390/vaccines13121189 (PMC12737558; doi:10.3390/vaccines13121189)
Supplement: Supplementary file 1 [file vaccines-13-01189-s001.zip › vaccines-3971755-supplementary.pdf]

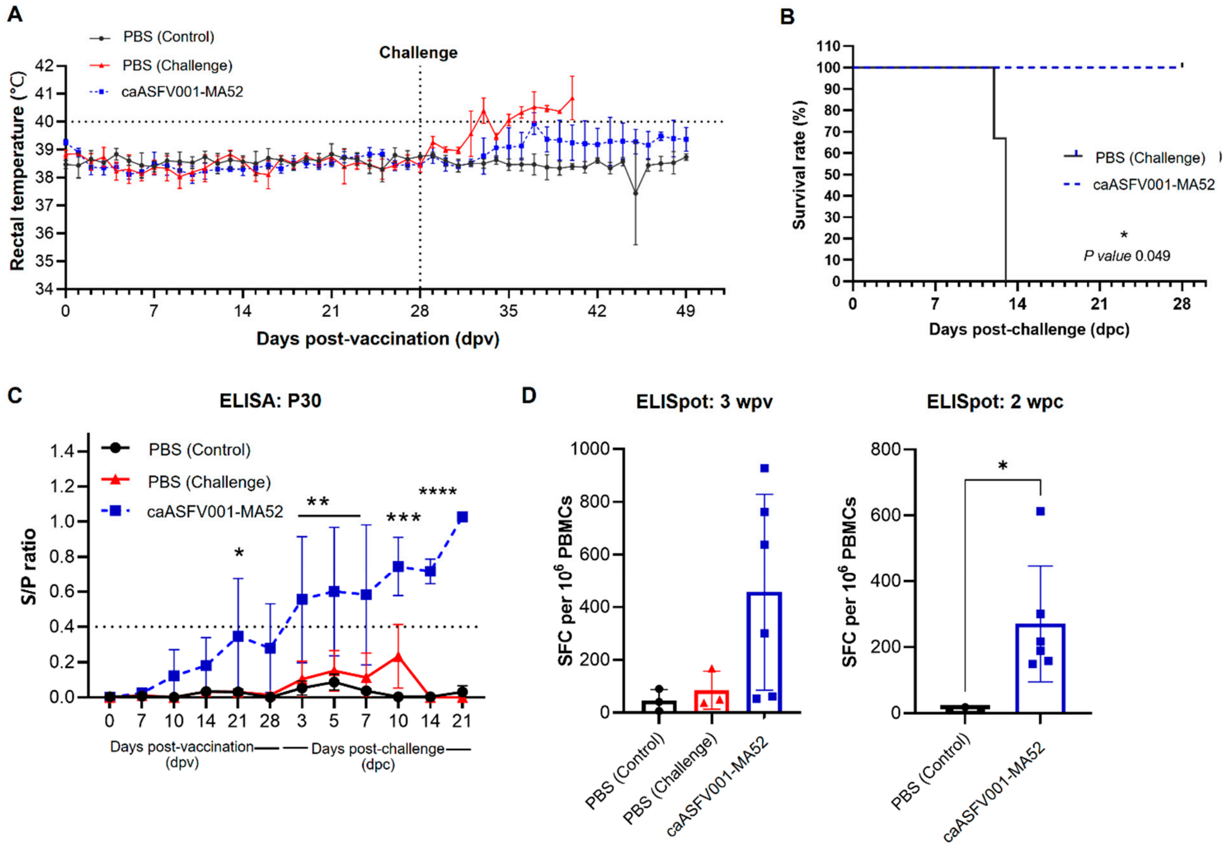

Figure S1. Preliminary challenge study of caASFV001-MA52 at a dose of 10^6 TCID$_{50}$. Seven-week-old pigs were separated into three experimental groups. The negative control group (n=3), PBS (Control), was given PBS and not challenged. The positive control group (n=3), PBS (Challenge), was given PBS and challenged with ASFV/GII/Thailand/NIAH-CR-19/2022 at a lethal dose of $10^{0.5}$ TCID$_{50}$. The test group (n=6), caASFV001-MA52, was given the prototype vaccine at a dose of 10^6 TCID$_{50}$ and subsequently challenged. (A) Body temperatures were measured rectally at the indicated time points and averaged for each group. (B) The survival rate of the two challenge groups was assessed post-challenge. Comparison of survival curves was determined using the Mantel–Cox log-rank test. (C) Blood samples were collected at the indicated time points and assessed for antibody by ELISA. Dashed line represents the cut-off value of S/P ratio. (D) PBMCs were harvested at 3 weeks post-vaccination (wpv) and 2 weeks post-challenge (wpc), stimulated with ASFV at an MOI of 0.1, and specific anti-ASFV responses were measured by counting the number of spots on the membrane of the porcine IFN- γ ELISpot assay. Results are expressed as spots per 10^6 PBMCs. Data was assessed using a two-way ANOVA, followed by Tukey's multiple comparisons test to evaluate differences between treatment groups. Error bars represent means ± SD. **** $p < 0.0001$, ***$p < 0.001$, **$p < 0.01$, *$p < 0.05$.

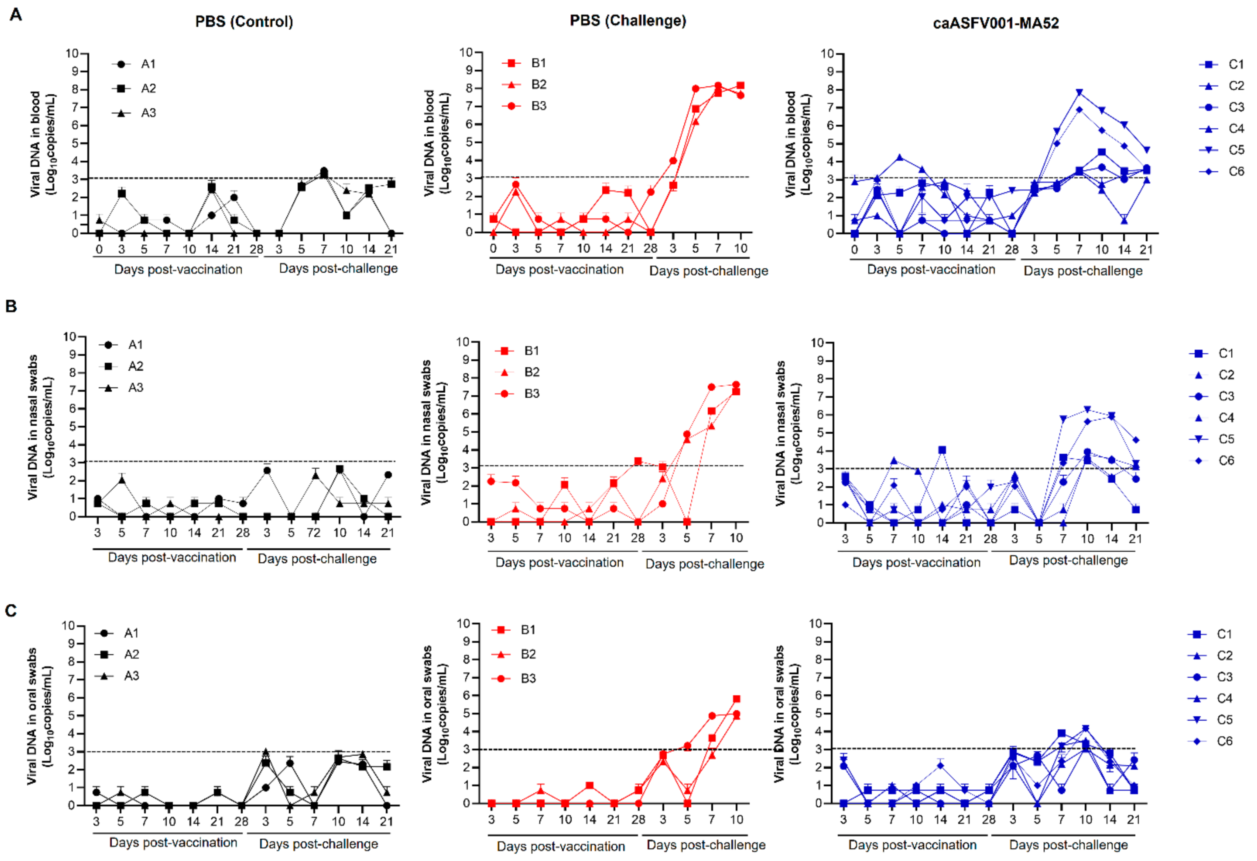

Figure S2. ASFV DNA levels in various samples of individual pig in the preliminary challenge study of caASFV001-MA52 at a dose of 10^6 TCID$_{50}$. Seven-week-old pigs were separated into three experimental groups. The negative control group (*n*=3), PBS (Control), was given PBS and not challenged. The positive control group (*n*=3), PBS (Challenge), was given PBS and challenged with ASFV/GII/Thailand/NIAH-CR-19/2022 at a lethal dose of $10^{0.5}$ TCID$_{50}$. The test group (*n*=6), caASFV001-MA52, was given the prototype vaccine at a dose of 10^6 TCID$_{50}$ and subsequently challenged. (A) Blood samples, (B) nasal swabs, and (C) oral swabs were collected at various time points and analyzed for the presence of ASFV DNA by qPCR. The dashed line represents the limit of detection of the qPCR assay

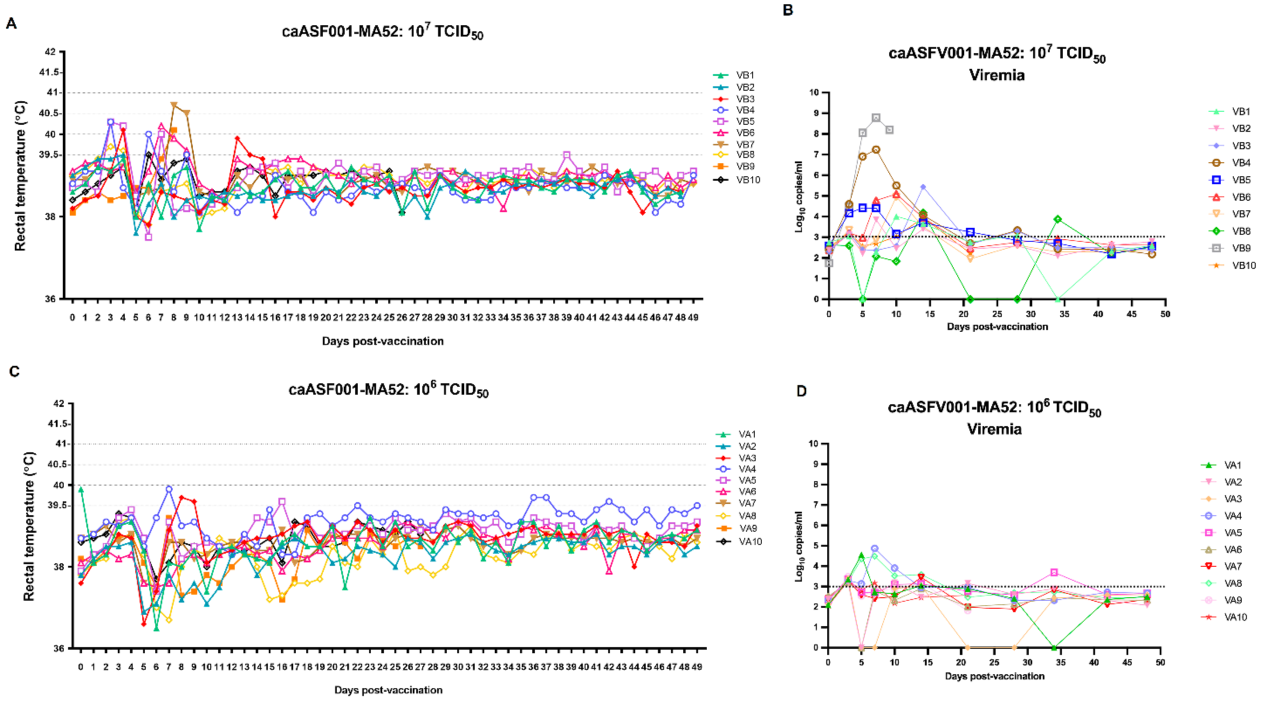

Figure S3. Preliminary safety study of caASFV001-MA52 at a dose of 10^6 $TCID_{50}$ and 10^7 $TCID_{50}$. Seven-week-old pigs were separated into 2 experimental groups (10 pigs per group) which received either caASFV001-MA52 at a dose of 10^7 and 10^6 $TCID_{50}$. (A, C) Rectal temperature of individual pigs was monitored daily and (B, D) blood samples of individual pigs were collected at various time points and analyzed for the presence of ASFV DNA by qPCR. The dashed line represents the limit of detection of the qPCR assay

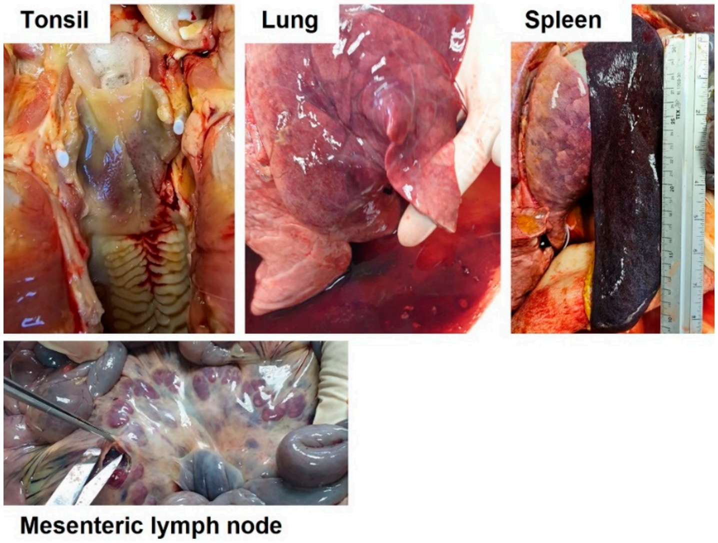

Figure S4. Gross pathological findings of vaccinated pig at necropsy. Pigs were given caASFV001-MA52 at a dose of 10^7 $TCID_{50}$ in the safety trial. One animal succumbed (VB9) at 18 days post-vaccination. Upon necropsy, severe inflammation and severe congestion in several organs are shown.

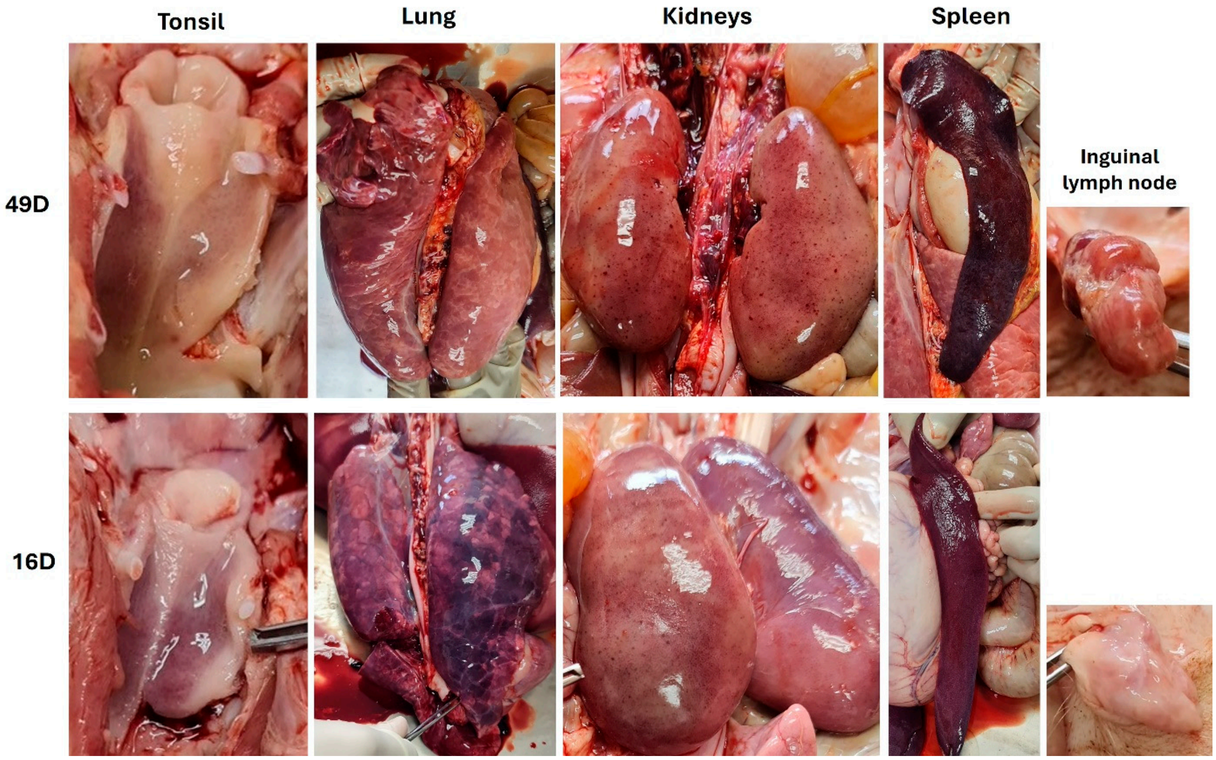

Figure S5. Gross pathological findings in pigs 49D and 16D. Group D[10^5] received 10^5 $TCID_{50}$ of caASFV001-MA52. Pigs 49D and 16D succumbed at 13 and 16 days post-vaccination respectively, and necropsy was performed. Tonsils, lungs, kidneys, spleens and inguinal lymph nodes are shown. Hemorrhaging was observed in various organs, severe pneumonia in the lung, and swollen, reddened inguinal lymph node. Pig 49D had splenomegaly with areas of infarction characterized by hemorrhage and ischemic necrosis.

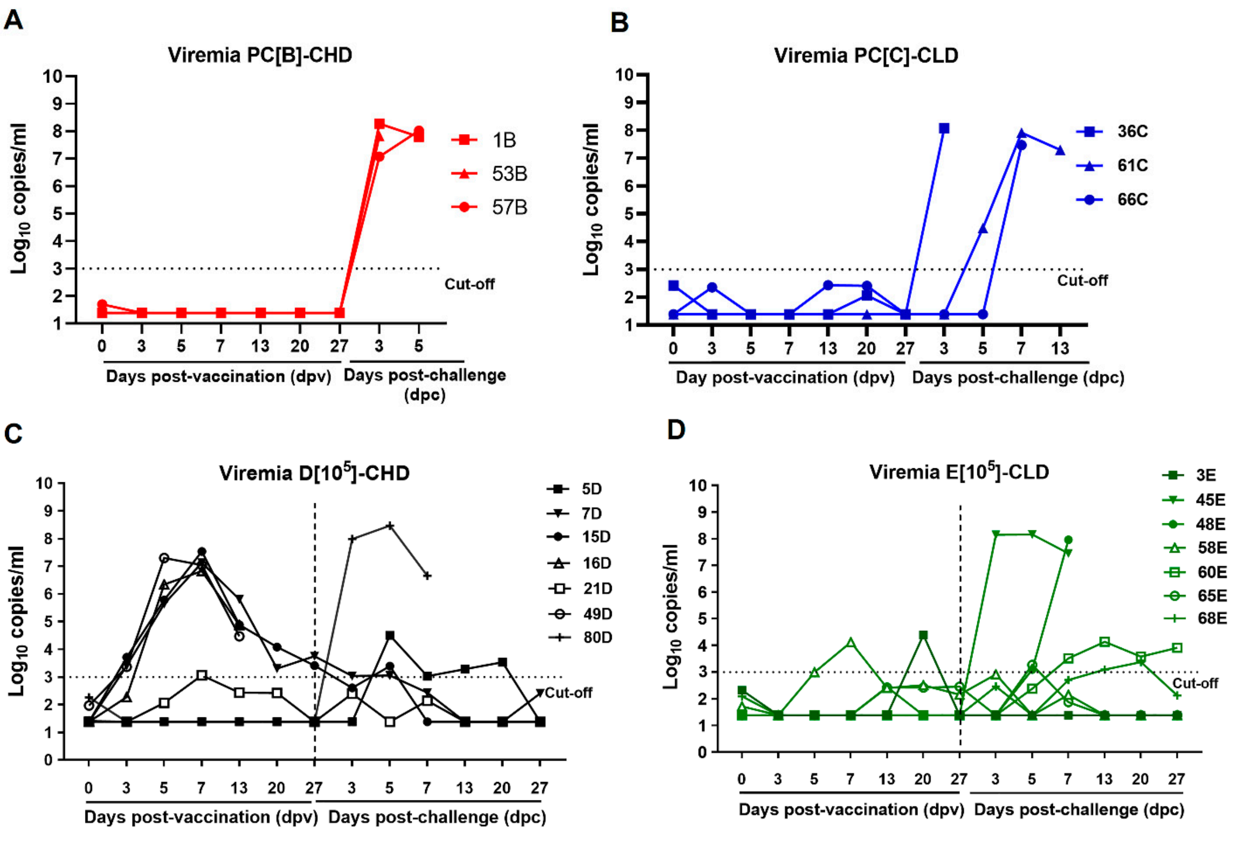

Figure S6. ASFV DNA levels in blood samples of individual pigs pre- and post-challenge. (A) Negative control (NC), (B) PC[B]-CHD and (C) PC[C]-CLD received PBS. NC was not challenged, while PC[B]-CHD and PC[C]-CLD were challenged at a dose of 10^2 and $10^{0.5}$ $TCID_{50}$ of the challenge virus, respectively. (D) Group D[10^5]-CHD received 10^5 $TCID_{50}$ of caASFV001-MA52 and 10^2 $TCID_{50}$ of the challenge virus. (E) Group E[10^5]-CLD received 10^5 $TCID_{50}$ of caASFV001-MA52 and $10^{0.5}$ $TCID_{50}$ of the challenge virus. Blood samples were collected at various time points before the challenge and analyzed for the presence of ASFV DNA by qPCR. Viral DNA copy numbers are shown for individual pigs in each group.

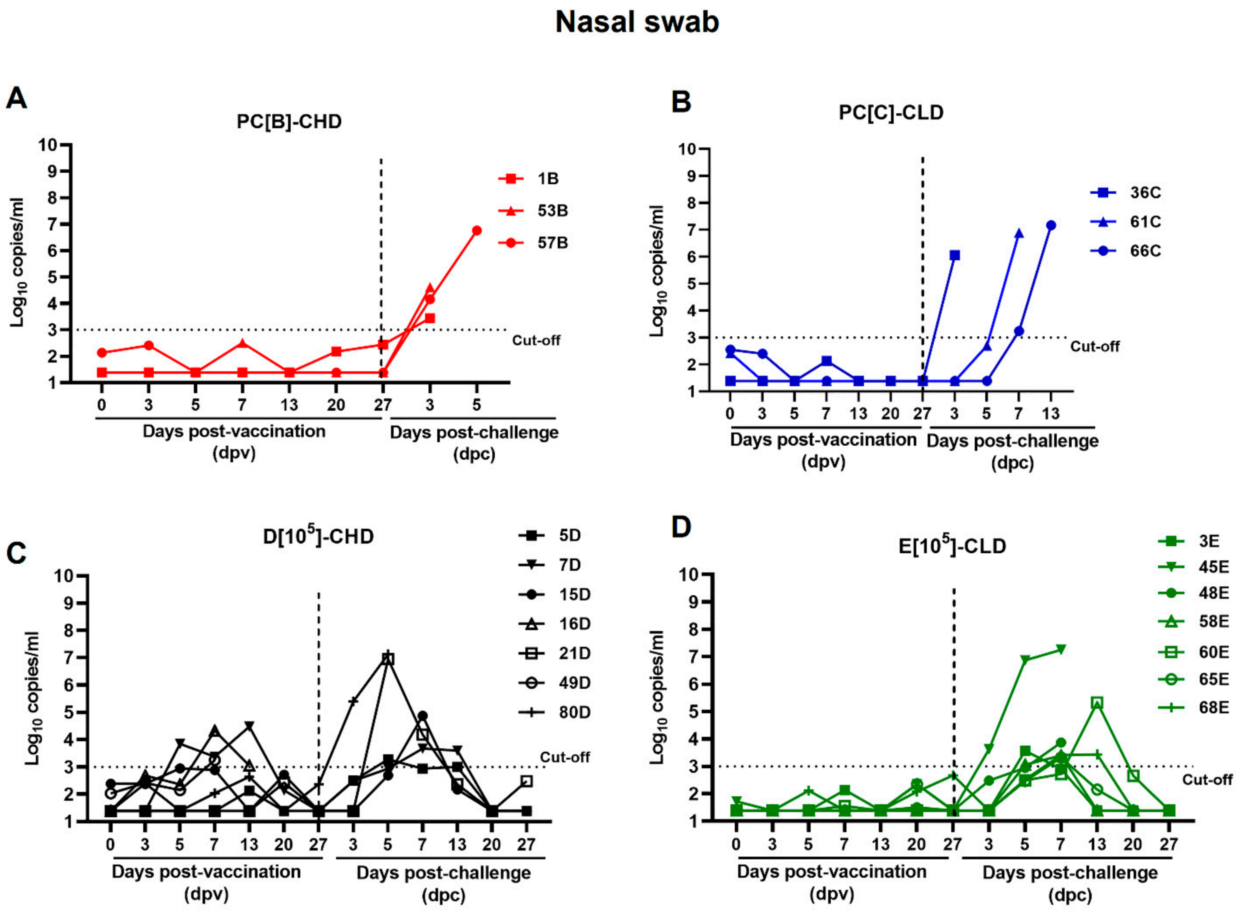

Figure S7. **ASFV DNA levels in nasal swabs throughout the experimental trial.** (A) Negative control (NC), (B) PC[B]-CHD and (C) PC[C]-CLD received PBS. NC was not challenged, while PC[B]-CHD and PC[C]-CLD were challenged at a dose of 10^2 and $10^{0.5}$ $TCID_{50}$ of the challenge virus, respectively. (D) Group D[10^5]-CHD received 10^5 $TCID_{50}$ of caASFV001-MA52 and 10^2 $TCID_{50}$ of the challenge virus. (E) Group E[10^5]-CLD received 10^5 $TCID_{50}$ of caASFV001-MA52 and $10^{0.5}$ $TCID_{50}$ of the challenge virus. Nasal swabs were collected at various time points before the challenge and analyzed for the presence of ASFV DNA by qPCR. Viral DNA copy numbers are shown for individual pigs in each group. The dashed line represents the limit of detection of the qPCR assay

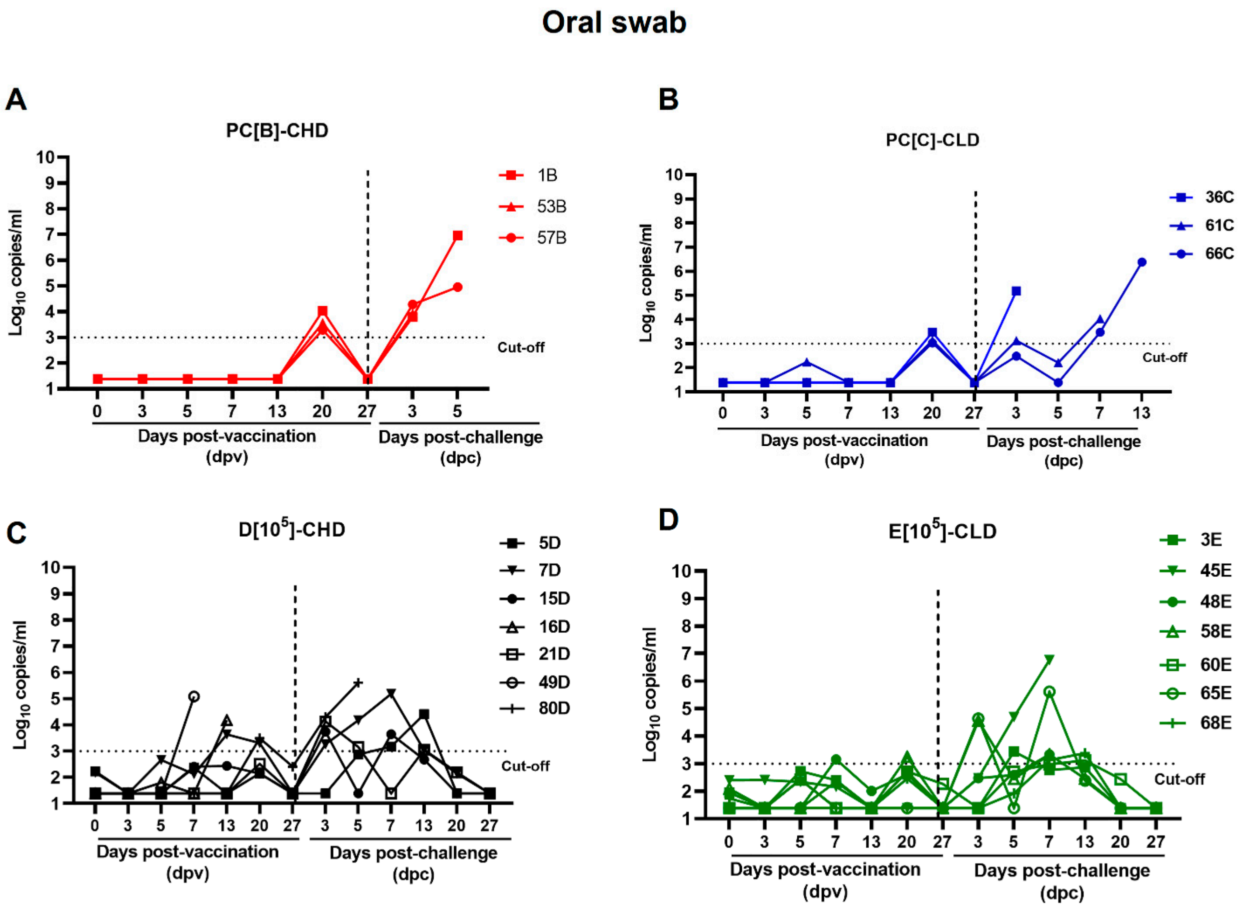

Figure S8. **ASFV DNA levels in oral swabs throughout the experimental trial.** (A) Negative control (NC), (B) PC[B]-CHD and (C) PC[C]-CLD received PBS. NC was not challenged, while PC[B]-CHD and PC[C]-CLD were challenged at a dose of 10^2 and $10^{0.5}$ $TCID_{50}$ of the challenge virus, respectively. (D) Group D[10^5]-CHD received 10^5 $TCID_{50}$ of caASFV001-MA52 and 10^2 $TCID_{50}$ of the challenge virus. (E) Group E[10^5]-CLD received 10^5 $TCID_{50}$ of caASFV001-MA52 and $10^{0.5}$ $TCID_{50}$ of the challenge virus. Oral swabs were collected at various time points before the challenge and analyzed for the presence of ASFV DNA by qPCR. Viral DNA copy numbers are shown for individual pigs in each group. The dashed line represents the limit of detection of the qPCR assay

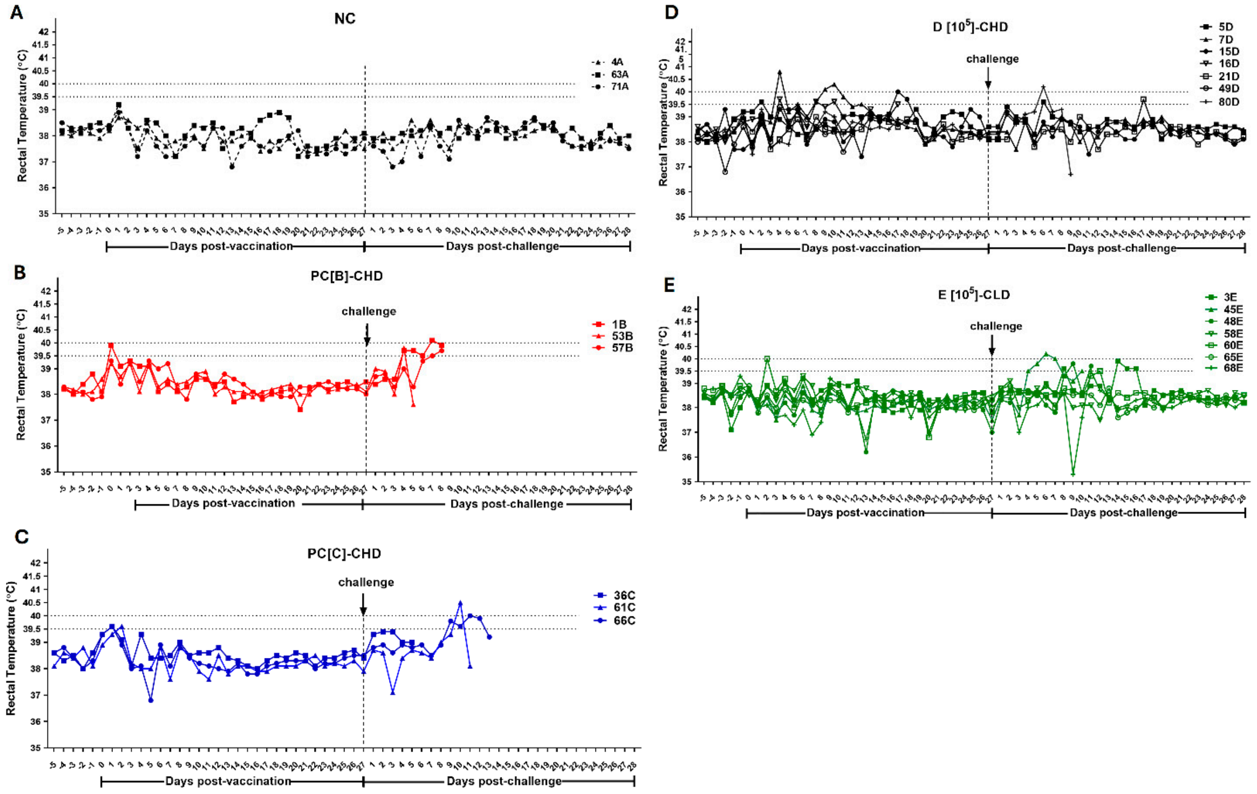

Figure S9. Rectal temperatures of individual pigs pre- and post-challenge. (A) Negative control (NC), (B) PC[B]-CHD and (C) PC[C]-CLD received PBS. NC was not challenged, while PC[B]-CHD and PC[C]-CLD were challenged at a dose of 10^2 and $10^{0.5}$ $TCID_{50}$ of the challenge virus, respectively. (D) Group D[10^5]-CHD received 10^5 $TCID_{50}$ of caASFV001-MA52 and 10^2 $TCID_{50}$ of the challenge virus. (E) Group E[10^5]-CLD received 10^5 $TCID_{50}$ of caASFV001-MA52 and $10^{0.5}$ $TCID_{50}$ of the challenge virus. Rectal temperatures were measured at the indicated time points and shown for individual pigs in in each group.

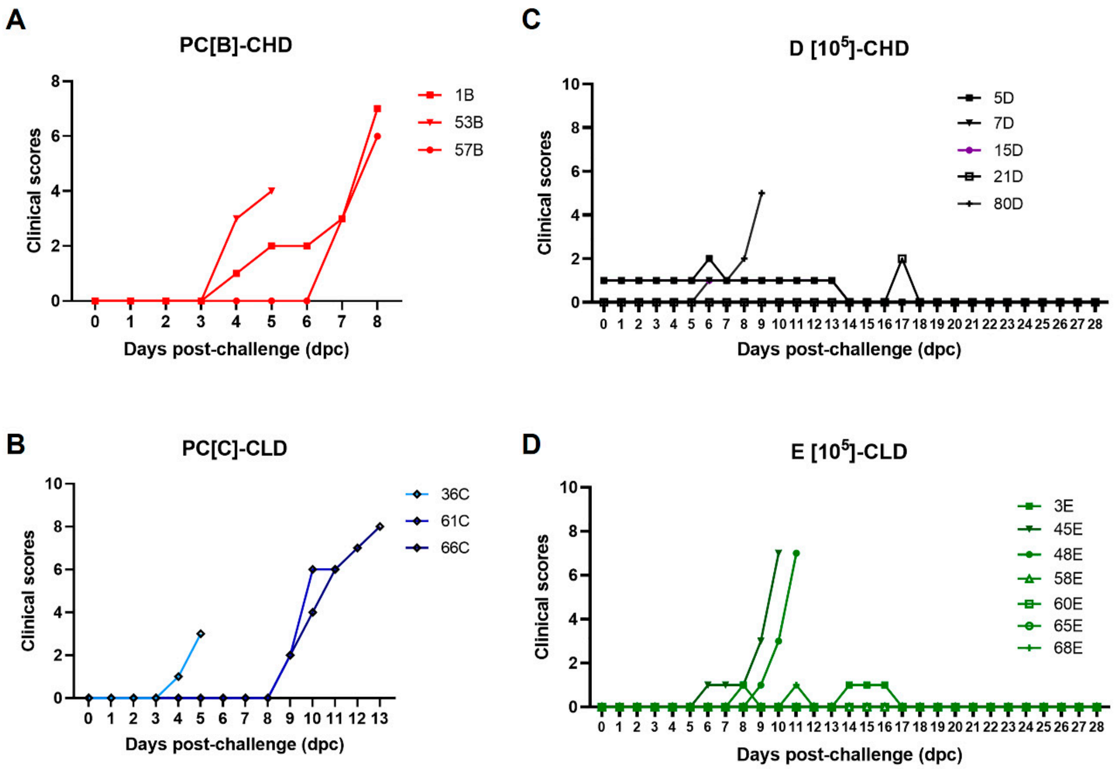

Figure S10. Daily cumulative clinical scores of individual pigs after challenge. Each symbol represents individual values for each animal in either group. PC[B]-CHD and PC[C]-CLD were given PBS and challenged with the virulent ASFV at a dose of 10^2 and $10^{0.5}$ $TCID_{50}$, respectively. Group D[10^5]-CHD received 10^5 $TCID_{50}$ of caASFV001-MA52 and 10^2 $TCID_{50}$ of the challenge virus. Group E[10^5]-CLD received 10^5 $TCID_{50}$ of caASFV001-MA52 and $10^{0.5}$ $TCID_{50}$ of the challenge virus.

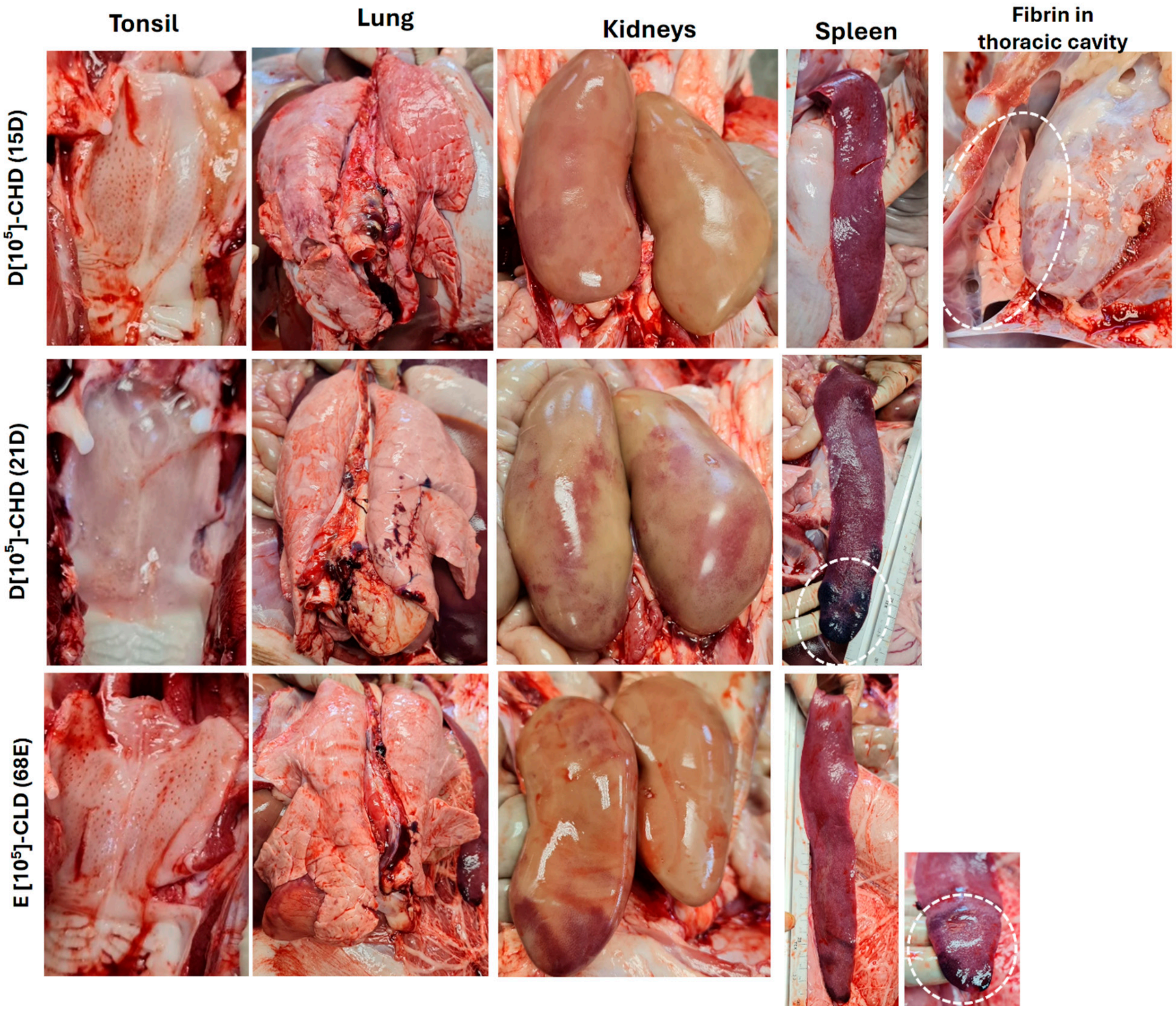

Figure S11. Gross pathological findings of the survivors at necropsy. Representative images of tonsils, lungs, kidneys, and spleens of 15D, 21D and 68E. In pig 15D, the presence of fibrin in the thoracic cavity is shown in the white circle. Group D[10^5]-CHD received 10^5 $TCID_{50}$ of caASFV001-MA52 and 10^2 $TCID_{50}$ of the challenge virus. Group E[10^5]-CLD received 10^5 $TCID_{50}$ of caASFV001-MA52 and $10^{0.5}$ $TCID_{50}$ of the challenge virus.

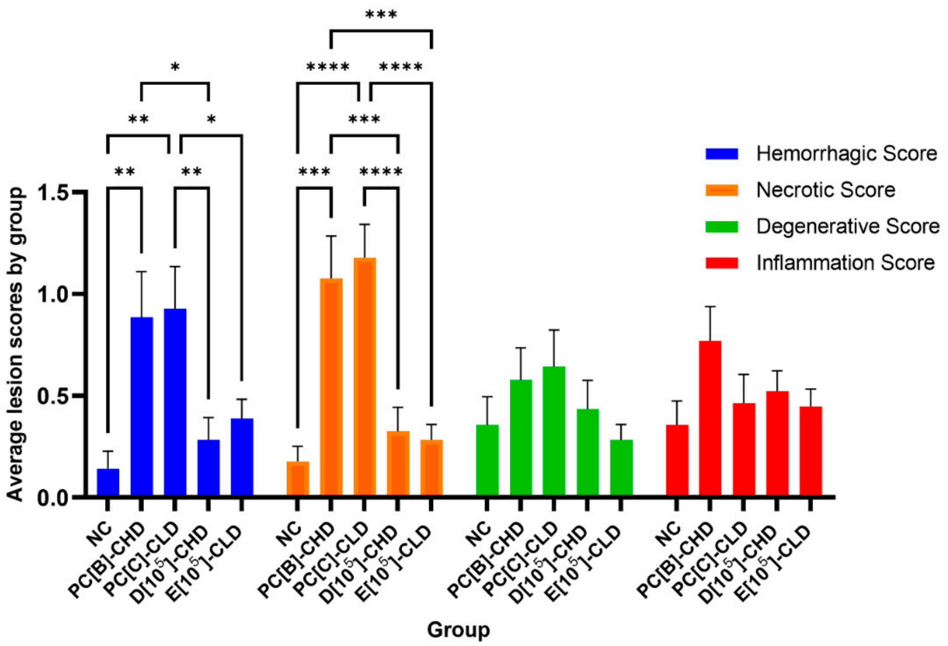

Figure S12. Average total lesion scores of controls and surviving pigs. H&E-stained tissues were scored for lesions for each pig that survived challenge. **A**verage total lesion scores in each tissue were calculated compared to each group. Error bars indicated means of total lesion scores ± standard deviation (SD). The difference between groups were calculated using the Two-way ANOVA and Turkey's multiple comparison test.*p < 0.05, **p < 0.01, ***p < 0.001, ****p < 0.0001. Negative control or NC, PC[B]-CHD and PC[C]-CLD were given PBS. NC was not challenged, while PC[B]-CHD and PC[C]-CLD were challenged at a dose of 10^2 and $10^{0.5}$ $TCID_{50}$, respectively. Group D[10^5]-CHD received 10^5 $TCID_{50}$ of caASFV001-MA52 and 10^2 $TCID_{50}$ of the challenge virus. Group E[10^5]-CLD received 10^5 $TCID_{50}$ of caASFV001-MA52 and $10^{0.5}$ $TCID_{50}$ of the challenge virus.

Table S1. PCR screening of viral and bacterial pathogens in serum samples from pigs on the farm prior to entering the experimental facility (1st screening)

Pig numbers	qPCR									Conventional PCR
	PCV		PRRS			ASFV	Mhr	Mhp	CSFV	*Streptococcus suis*
	PCV2	PCV3	EU	US	HP					
1	-	-	-	-	-	-	-	-	-	-
2	-	-	-	-	-	-	-	-	-	-
3	+ (ct 35.63)	-	-	-	-	-	-	-	-	-
4	-	-	-	-	-	-	-	-	-	-
5	-	-	-	-	-	-	-	-	-	-
6	+ (ct 31.94)	+ (ct 35.87)	-	-	-	-	+ (ct 37.12)	+ (ct 36.08)	-	-
7	-	-	-	-	-	-	-	-	-	-
8	-	-	-	-	-	-	+ (ct 34.53)	+ (ct 37.25)	-	-
9	-	-	-	-	-	-	+ (ct 35.68)	+ (ct 34.75)	-	-
10	-	-	-	-	-	-	+ (ct 35.53)	-	-	-
11	-	-	-	-	-	-	+ (ct 36.62)	-	-	-
12	+ (ct 34.48)	+ (ct 38.17)	-	-	-	-	+ (ct 37.53)	-	-	-
13	-	-	-	-	-	-	-	-	-	-
14	-	-	-	-	-	-	+ (ct 35.35)	+ (ct 36.63)	-	-
15	-	-	-	-	-	-	-	-	-	-
16	-	-	-	-	-	-	-	-	-	-
17	-	-	-	-	-	-	+ (ct 36.36)	+ (ct 33.58)	-	-
18	-	-	-	-	-	-	+ (ct 36.45)	-	-	-
19	-	-	-	-	-	-	+ (ct 35.27)	-	-	-
20	-	-	-	-	-	-	-	+ (ct 36.27)	-	-
21	-	-	-	-	-	-	-	-	-	-
22	+ (ct 35.36)	+ (ct 37.93)	-	-	-	-	+ (ct 36.70)	+ (ct 37.14)	-	-
23	+ (ct 29.14)	+ (ct 31.79)	-	-	-	-	+ (ct 34.36)	-	-	-
24	-	-	-	-	-	-	+ (ct 36.03)	-	-	-
25	-	-	-	-	-	-	+ (ct 35.91)	+ (ct 35.00)	-	-
26	-	-	-	-	-	-	+ (ct 36.87)	+ (ct 35.43)	-	-
27	-	-	-	-	-	-	+ (ct 36.58)	-	-	-
28	-	-	-	-	-	-	+ (ct 36.92)	+ (ct 37.59)	-	-
29	-	-	-	-	-	-	-	-	-	-
30	-	-	-	-	-	-	+ (ct 36.76)	-	-	-
31	+ (ct 26.50)	+ (ct 29.28)	-	-	-	-	+ (ct 34.08)	+ (ct 36.04)	-	-
32	-	-	-	-	-	-	+ (ct 37.77)	+ (ct 37.34)	-	-
33	-	-	-	-	-	-	+ (ct 34.67)	-	-	-
34	-	-	-	-	-	-	+ (ct 36.15)	+ (ct 35.71)	-	-
35	-	-	-	-	-	-	-	+ (ct 36.09)	-	-
36	-	-	-	-	-	-	-	-	-	-
37	+ (ct 36.18)	+ (ct 38.47)	-	-	-	-	+ (ct 36.03)	-	-	-
38	-	-	-	-	-	-	+ (ct 35.89)	-	-	-
39	-	-	-	-	-	-	+ (ct 36.18)	-	-	-
40	-	-	-	-	-	-	+ (ct 35.55)	-	-	-

Table S2. Anti-ASFV antibody levels in pig serum samples prior to entering the experimental facility.

Pig numbers	PCR screening results	ELISA	
		S/P ratio	Interpretation
1	All negative	0.60	Negative
2	All negative	0.68	Negative
3	All negative	2.31	Negative
4	All negative	2.99	Negative
5	All negative	0.42	Negative
7	All negative	0.51	Negative
15	All negative	0.68	Negative
16	All negative	0.42	Negative
21	All negative	0.34	Negative
29	All negative	6.42	Negative
36	All negative	0.51	Negative
42	All negative	0.42	Negative
45	All negative	0.42	Negative
46	All negative	0.51	Negative
47	All negative	1.28	Negative
48	All negative	2.14	Negative
49	All negative	1.54	Negative
50	All negative	0.60	Negative
52	All negative	1.20	Negative
53	All negative	1.54	Negative
58	All negative	0.34	Negative
61	All negative	0.42	Negative
63	All negative	0.85	Negative
64	All negative	0.42	Negative
65	All negative	0.42	Negative
66	All negative	0.68	Negative
67	All negative	1.79	Negative
68	All negative	0.85	Negative
71	All negative	0.42	Negative
80	All negative	0.77	Negative

Table S3. PCR screening of viral and bacterial pathogens in serum samples of individual pigs (0 days post-vaccination: dpv)

No.	Groups	Pig numbers	qPCR									Conventional PCR
			PCV		PRRS			ASFV	Mhr	Mhp	CSFV	*Streptococcus suis*
			PCV2	PCV3	EU	US	HP					
**1**	A	4	-	-	-	-	-	-	-	-	-	-
**2**		63	-	-	-	-	-	-	-	-	-	-
**3**		71	-	-	-	-	-	-	-	-	-	-
**4**	B	1	-	-	-	+(ct 43.68)	-	-	-	-	-	-
**5**		53	-	-	-	+(ct 42.32)	-	-	-	-	-	-
**6**		57	+(ct 30.79)	+(ct 30.38)	-	-	-	-	-	-	-	-
**7**	C	36	+(ct 40.25)	+(ct 38.60)	-	-	-	-	-	-	-	-
**8**		61	-	-	-	-	+(ct 38.53)	-	-	-	-	-
**9**		66	-	-	-	-	+(ct 38.74)	-	-	-	-	-
**10**	D	5	-	-	-	-	-	-	-	-	-	-
**11**		7	-	-	-	-	-	-	-	-	-	-
**12**		15	-	-	-	-	-	-	-	-	-	-
**13**		16	-	-	-	-	-	-	-	-	-	-
**15**		21	-	-	-	-	-	-	-	-	-	-
**16**		49	-	+(ct 40.44)	-	-	-	-	-	-	-	-
**17**		80	-	-	-	-	-	-	-	-	-	-
**18**	E	3	-	-	-	-	-	-	-	-	-	-
**19**		45	-	-	-	-	-	-	-	-	-	-
**20**		48	-	-	-	-	-	-	-	-	-	-
**21**		58	-	-	-	-	-	-	-	-	-	-
22		60	-	-	-	-	-	-	-	-	-	-
23		65	-	-	-	-	-	-	-	-	-	-
24		68	-	-	-	-	-	-	-	-	-	-

Table S4. PCR screening of viral and bacterial pathogens in serum samples of individual pigs (7 days post-vaccination: dpv)

No.	Groups	Pig numbers	qPCR									Conventional PCR
			PCV		PRRS			ASFV	Mhr	Mhp	CSFV	*Streptococcus suis*
			PCV2	PCV3	EU	US	HP					
1	A	4	-	-	+ (ct 38.85)	-	-	-	-	-	-	-
2		63	-	-	+ (ct 42.14)	-	-	-	-	-	-	-
3		71	-	-	-	-	-	-	-	-	-	-
4	B	1	-	-	-	-	-	-	-	-	-	-
5		53	-	-	-	-	-	-	-	-	-	-
6		57	+ (ct 30.23)	+ (ct 28.63)	-	-	-	-	-	-	-	-
7	C	36	-	-	-	-	-	-	-	-	-	-
8		61	-	-	+ (ct 39.02)	-	-	-	-	-	-	-
9		66	-	-	-	-	-	-	-	-	-	-
10	D	5	-	-	-	-	-	-	-	-	-	-
11		7	+ (ct 37.59)	-	-	+ (ct 40.5)	-	+ (ct 26.43)	-	-	-	-
12		15	+ (ct 37.79)	+ (ct 37.57)	-	-	-	+ (ct 25.85)	-	-	-	-
13		16	-	-	-	-	-	+ (ct 27.33)	-	-	-	-
15		21	-	-	-	-	-	-	-	-	-	-
16		49	-	-	-	-	-	+ (ct 23.66)	-	-	-	-
17		80	-	-	-	-	-	-	-	-	-	-
18	E	3	-	-	-	-	-	-	-	-	-	-
19		45	-	-	+ (ct 39.81)	+ (ct 40.83)	-	-	-	-	-	-
20		48	-	-	-	-	-	-	-	-	-	-
21		58	+ (ct 38.24)	-	-	-	-	+ (ct 34.64)	-	-	-	-
22		60	-	-	+ (ct 40.44)	-	-	-	-	-	-	-
23		65	+ (ct 37.33)	+ (ct 38.08)	-	-	-	-	-	-	-	-
24		68	+ (ct 36.02)	+ (ct 36.69)	-	-	-	-	-	-	-	-
	feces	5D	+ (ct 37.72)	-	-	-	-	+ (ct 38.06)	-	-	-	+
		49D	-	+ (ct 39.26)	-	-	-	+ (ct 23.62)	-	-	-	+
		45E	+ (ct 38.05)	-	-	-	-	-	-	-	-	-

Table S5. PCR screening of viral and bacterial pathogens in serum samples of individual pigs (13 days post-vaccination: dpv)

No.	Groups	Pig numbers	qPCR									Conventional PCR
			PCV		PRRS			ASFV	Mhr	Mhp	CSFV	*Streptococcus suis*
			PCV2	PCV3	EU	US	HP					
1		4	-	-	-	-	-	-	-	-	-	-
2	A	63	-	-	+ (ct 38.63)	-	-	-	-	-	-	-
3		71	-	-	+ (ct 38.57)	-	-	-	-	-	-	-
4		1	-	-	-	-	-	-	-	-	-	-
5	B	53	-	-	-	-	-	-	-	-	-	-
6		57	+ (ct 28.49)	+ (ct 26.31)	-	-	-	-	-	-	-	-
7		36	-	-	-	-	-	-	-	-	-	-
8	C	61	-	-	-	+ (ct 42.46)	-	-	-	-	-	-
9		66	-	+ (ct 39.62)	-	+ (ct 43.02)	-	-	-	-	-	-
10		5	-	-	+ (ct 37.7)	-	-	-	-	-	-	-
11		7	-	-	-	-	-	-	-	-	-	-
12		15	-	-	-	-	-	-	-	-	-	-
13	D	16	-	-	-	-	-	+ (ct 32.43)	-	-	-	-
15		21	-	-	+ (ct 37.48)	-	-	-	-	-	-	-
16		49	-	-	-	-	-	+ (ct 30.82)	-	-	-	-
17		80	-	-	-	-	-	-	-	-	-	-
18		3	-	-	-	-	-	-	-	-	-	-
19		45	-	-	-	-	-	-	-	-	-	-
20		48	-	-	-	-	-	-	-	-	-	-
21	E	58	-	-	-	-	-	-	-	-	-	-
22		60	-	-	-	-	-	-	-	-	-	-
23		65	-	-	-	-	-	-	-	-	-	-
24		68	-	-	-	-	-	-	-	-	-	-

Table S6. PCR screening of viral and bacterial pathogens in serum samples of individual pigs (20 days post-vaccination: dpv)

No.	Groups	Pig numbers	qPCR									Conventional PCR
			PCV		PRRS			ASFV	Mhr	Mhp	CSFV	*Streptococcus suis*
			PCV2	PCV3	EU	US	HP					
**1**	A	4	-	-	-	-	-	-	-	-	-	-
**2**		63	-	-	-	-	-	-	-	-	-	-
**3**		71	-	-	-	-	-	-	-	-	-	-
**4**	B	1	-	-	-	-	-	-	-	-	-	-
**5**		53	-	-	-	+ (ct 40.26)	-	-	-	-	-	-
**6**		57	+ ct34.72)	+ ct30.54)	-	-	-	-	-	-	-	-
**7**	C	36	-	-	-	-	-	-	-	-	-	-
**8**		61	-	-	-	-	-	-	-	-	-	-
**9**		66	-	-	-	-	-	-	-	-	-	-
**10**	D	5	-	-	-	-	-	-	-	-	-	-
**11**		7	-	-	-	-	-	-	-	-	-	-
**12**		15	-	-	-	-	-	-	-	-	-	-
**13**		16	-	-	-	-	-	+(ct 39.64)	-	-	-	-
**15**		21	-	-	-	-	-	-	-	-	-	-
**16**		80	-	-	-	+ (ct 40.28)	-	-	-	-	-	-
**18**	E	3	-	-	-	-	-	-	-	-	-	-
**19**		45	-	-	-	-	-	-	-	-	-	-
**20**		48	-	-	-	-	-	-	-	-	-	-
**21**		58	-	-	-	-	-	-	-	-	-	-
22		60	-	-	-	-	-	-	-	-	-	-
23		65	-	-	-	+ (ct 36.5)	-	-	-	-	-	-
24		68	-	-	-	-	-	-	-	-	-	-

Table S7. PCR screening of viral and bacterial pathogens in serum samples of individual pigs (27 days post-vaccination: dpv)

No.	Groups	Pig numbers	qPCR									Conventional PCR
			PCV		PRRS			ASFV	Mhr	Mhp	CSFV	*Streptococcus suis*
			PCV2	PCV3	EU	US	HP					
**1**	A	4	-	-	-	-	-	-	-	-	-	-
**2**		63	-	-	-	-	-	-	-	-	-	-
**3**		71	-	-	-	-	-	-	-	-	-	-
**4**	B	1	-	-	-	-	-	-	-	-	-	-
**5**		53	-	-	-	+ (ct 40.26)	-	-	-	-	-	-
**6**		57	+ ct32.05)	+ ct29.37)	-	-	-	-	-	-	-	-
**7**	C	36	-	-	-	-	-	-	-	-	-	-
**8**		61	-	-	-	-	-	-	-	-	-	-
**9**		66	-	-	-	-	-	-	-	-	-	-
**10**	D	5	-	-	-	-	-	-	-	-	-	-
**11**		7	-	-	-	-	-	+(ct 36.55)	-	-	-	-
**12**		15	-	-	-	+ (ct 35.06)	-	-	-	-	-	-
**13**		16	-	-	-	-	-	+(ct 39.64)	-	-	-	-
**15**		21	-	-	-	+ (ct 40.85)	-	-	-	-	-	-
**16**		80	-	-	-	-	-	-	-	-	-	-
**18**	E	3	-	-	-	-	-	-	-	-	-	-
**19**		45	-	-	-	-	-	-	-	-	-	-
**20**		48	-	-	-	-	-	-	-	-	-	-
**21**		58	-	-	-	-	-	-	-	-	-	-
22		60	-	-	-	-	-	-	-	-	-	-
23		65	-	-	-	-	-	-	-	-	-	-
24		68	-	-	-	-	-	-	-	-	-	-
